# Histone H2B-IFI16 Recognition of Nuclear Herpesviral Genome Induces Cytoplasmic Interferon-β Responses

**DOI:** 10.1371/journal.ppat.1005967

**Published:** 2016-10-20

**Authors:** Jawed Iqbal, Mairaj Ahmed Ansari, Binod Kumar, Dipanjan Dutta, Arunava Roy, Leela Chikoti, Gina Pisano, Sujoy Dutta, Shahrooz Vahedi, Mohanan Valiya Veettil, Bala Chandran

**Affiliations:** H. M. Bligh Cancer Research Laboratories, Department of Microbiology and Immunology, Chicago Medical School, Rosalind Franklin University of Medicine and Science, North Chicago, Illinois, United States of America; Oregon Health and Science University, UNITED STATES

## Abstract

IFI16 (gamma-interferon-inducible protein 16), a predominantly nuclear protein involved in transcriptional regulation, also functions as an innate immune response DNA sensor and induces the IL-1β and antiviral type-1 interferon-β (IFN-β) cytokines. We have shown that IFI16, in association with BRCA1, functions as a sequence independent nuclear sensor of episomal dsDNA genomes of KSHV, EBV and HSV-1. Recognition of these herpesvirus genomes resulted in IFI16 acetylation, BRCA1-IFI16-ASC-procaspase-1 inflammasome formation, cytoplasmic translocation, and IL-1β generation. Acetylated IFI16 also interacted with cytoplasmic STING and induced IFN-β. However, the identity of IFI16 associated nuclear proteins involved in STING activation and the mechanism is not known. Mass spectrometry of proteins precipitated by anti-IFI16 antibodies from uninfected endothelial cell nuclear lysate revealed that histone H2B interacts with IFI16. Single and double proximity ligation microscopy, immunoprecipitation, EdU-genome labeled virus infection, and chromatin immunoprecipitation studies demonstrated that H2B is associated with IFI16 and BRCA1 in the nucleus in physiological conditions. *De novo* KSHV and HSV-1 infection as well as latent KSHV and EBV infection induces the cytoplasmic distribution of H2B-IFI16, H2B-BRCA1 and IFI16-ASC complexes. Vaccinia virus (dsDNA) cytoplasmic replication didn’t induce the redistribution of nuclear H2B-IFI16 or H2B into the cytoplasm. H2B is critical in KSHV and HSV-1 genome recognition by IFI16 during *de novo* infection. Viral genome sensing by IFI16-H2B-BRCA1 leads to BRCA1 dependent recruitment of p300, and acetylation of H2B and IFI16. BRCA1 knockdown or inhibition of p300 abrogated the acetylation of H2B-IFI16 or H2B. Ran-GTP protein mediated the translocation of acetylated H2B and IFI16 to the cytoplasm along with BRCA1 that is independent of IFI16-ASC inflammasome. ASC knockdown didn’t affect the acetylation of H2B, its cytoplasmic transportation, and the association of STING with IFI16 and H2B during KSHV infection. Absence of H2B didn’t affect IFI16-ASC association and cytoplasmic distribution and thus demonstrating that IFI16-H2B complex is independent of IFI16-ASC-procaspase-1-inflammasome complex formed during infection. The H2B-IFI16-BRCA1 complex interacted with cGAS and STING in the cytoplasm leading to TBK1 and IRF3 phosphorylation, nuclear translocation of pIRF3 and IFN-β production. Silencing of H2B, cGAS and STING inhibited IFN-β induction but not IL-1β secretion, and cGAMP activity is significantly reduced by H2B and IFI16 knockdown during infection. Silencing of ASC inhibited IL-1β secretion but not IFN-β secretion during *de novo* KSHV and HSV-1 infection. These studies identify H2B as an innate nuclear sensor mediating a novel extra chromosomal function, and reveal that two IFI16 complexes mediate KSHV and HSV-1 genome recognition responses, with recognition by the IFI16-BRCA1-H2B complex resulting in IFN-β responses and recognition by IFI16-BRCA1 resulting in inflammasome responses.

## Author Summary

Eukaryotic cells elicit innate immune responses against invading microbes including viruses. IFI16, a predominantly nuclear protein, has emerged as an innate response nuclear DNA sensor. Recognition of nuclear KSHV, HSV-1 and EBV dsDNA genomes by IFI16-BRCA1 leads to IFI16 acetylation, cytoplasmic translocation of the BRCA1-IFI16-ASC-procaspase-1 inflammasome complex and IL-1β generation. Here, we demonstrate that histone H2B is associated with IFI16-BRCA1 in the nucleus under physiological conditions. Recognition of nuclear viral genomes by IFI16-H2B-BRCA1 leads to BRCA1-p300 mediated acetylation of H2B and IFI16, and cytoplasmic transport of H2B-IFI16-BRCA1 via Ran GTP protein. The inflammasome independent cytoplasmic IFI16-H2B-BRCA1 complex interacts with cGAS and STING resulting in TBK1 and IRF3 phosphorylation, and nuclear pIRF3-mediated IFN-β induction. H2B knockdown inhibits IFN-β production while ASC silencing doesn’t affect IFN-β induction. Our studies identify H2B as an innate nuclear sensor and reveal that two IFI16 complexes mediate nuclear herpesviral genome recognition responses, IFI16-BRCA1-H2B-IFN-β responses and IFI16-BRCA1-inflammasome responses.

## Introduction

RNA and DNA genomes of viruses are recognized by several host innate immune response sensors in different subcellular locations, resulting in antiviral responses of type 1 interferon (IFN) and inflammasome activation [1]. We have shown that IFI16 (interferon inducible protein 16), a resident nuclear protein involved in transcriptional regulation by an unknown mechanism, also functions as a nuclear sensor of innate immune inflammasome and IFN-β responses [2–5]. IFI16 detects the nuclear replicating episomal herpesvirus genomes of Kaposi's sarcoma-associated herpesvirus (KSHV), Epstein-Barr virus (EBV), and herpes simplex virus type-1 (HSV-1). This leads to IFI16-ASC-procaspase-1 inflammasome formation in the nucleus, which is transported to the cytoplasm leading into caspase-1 activation and pro-IL-1β/IL-18 cleavages [2–6]. We and others have also shown that independent of ASC, KSHV and HSV-1 genome recognition results in IFI16 interaction with STING in the cytoplasm, phosphorylation and nuclear translocation of IRF3, IFN gene expression and IFN-β production [1, 4, 6–8].

Our recent studies have shown that BRCA1, a DNA damage response (DDR) sensor and transcription regulator, is in complex with IFI16 in the uninfected cell nucleus. This BRCA1-IFI16 interaction increased during *de novo* KSHV, EBV and HSV-1 infection and in cells latently infected with KSHV and EBV, but not by bleomycin induced DDR or by cytoplasmic dsDNA vaccinia virus replication [1]. BRCA1 is a constituent of the genome recognition triggered IFI16-inflammasome that translocates to the cytoplasm. IFI16’s recognition of KSHV and HSV-1 genomes was inhibited without BRCA1, demonstrating that sensing of viral DNA by IFI16 depends on its pre-existing complex with BRCA1. In the absence of BRCA1, the consequences of viral genome sensing, such as IFI16-inflammasome assembly, cytoplasmic localization, IL-1β production, and cytoplasmic IFI16-STING interaction, pIRF3 and IFN-β induction, were inhibited [1].

Our studies have also revealed that IFI16 undergoes acetylation by p300 (histone acetyl transferase) facilitating IFI16-ASC-procaspase-1 association, cytoplasmic translocation via Ran-GTP resulting in IL-1β production, and interaction with STING and IFN-β induction [9]. Leptomycin B treatment abrogated acetylated IFI16 translocation to the cytoplasm. ASC and STING knockdowns did not affect IFI16 acetylation demonstrating that this modification is upstream of inflammasome assembly and STING activation [9]. p300 inhibitor C646 or knockdown of p300 did not inhibit the association of IFI16 with KSHV and HSV-1 genomes signifying that increased nuclear acetylation of IFI16 is a post-nuclear genome recognition event that is common to IFI16-mediated inflammasome and IFN-β induction during KSHV, EBV, and HSV-1 infections [9].

Although IFI16 is the primary nuclear DNA sensor during HSV-1 infection, IFI16 interactions with cGAS (cGAMP-Synthase) and stabilization of IFI16 by cGAS have been reported [10]. cGAS senses cytosolic DNA leading to the production of second messenger cGAMP which activates STING to stimulate IFN-β production [11–14]. DNA damage induced leakage of self DNA into the cytoplasm has been shown to activate the IFN-β pathway [15], while extra-chromosomal cytoplasmic histone H2B is suggested to be involved in aberrant self-or non-self-dsDNA recognition and induction of IFN-β [16].

We observed earlier that IFN-β was induced in the absence of ASC [6] and acetylated IFI16 was still detected in the cytoplasm of KSHV infected cells although the total and acetylated IFI16 levels were reduced by >3-fold compared to the levels in the presence of ASC [9]. We hypothesized that the cytoplasmic redistribution of IFI16 in ASC knockdown cells must be an inflammasome independent event which might be attributed to cytoplasmic export of acetylated IFI16 either alone or in complex with other nuclear proteins resulting in the activation of STING. To identify the IFI16 associated nuclear proteins involved in STING activation, uninfected endothelial cell nuclear lysate was precipitated by anti-IFI16 antibodies. Mass spectrometry (MS) of specific protein bands revealed that histone H2B was interacting with IFI16.

Here, we demonstrate that H2B is an essential component of the post-KSHV and HSV-1 genome recognition induced IFI16-mediated IFN-β production. Viral genome recognition by IFI16 led to the BRCA1 dependent p300-IFI16 interaction, acetylation of H2B and IFI16 in the nucleus, and their export via Ran-GTP. H2B-IFI16 along with BRCA1 interacted with cGAS and STING in the cytoplasm, resulting in pIRF3 induction and IFN-β production. Knockdown of H2B impaired the IFI16-mediated IFN-β response during KSHV and HSV-1 *de novo* infection and did not affect IFI16-inflammasome induction. Cytoplasmic distribution of H2B-IFI16 is also observed in cells latently infected with EBV. Collectively, these studies demonstrate that H2B is a crucial component in herpesvirus nuclear genome sensing by IFI16 and in the consequent innate IFN-β response.

## Results

### IFI16 interacts with histone H2B in the uninfected cell nucleus

To determine the identity of IFI16 associated nuclear proteins potentially involved in cytoplasmic activation of STING in the absence of ASC, nuclear lysates from uninfected primary human microvascular dermal endothelial (HMVEC-d) cells were IP-ed with anti-IFI16 antibodies and specific bands were analyzed by MS. Among the identified IFI16 interacting proteins, histone H2B (~13 kDa) had the highest PEAKS score and coverage (S1 Table). This interested us since apart from its epigenetic roles in the nucleus, extrachromosomal cytoplasmic histone H2B has been shown to be involved in the induction of IFN-β against small DNA fragments [17]. However, how H2B translocated into the cytoplasm and whether H2B plays a role in innate responses against nuclear genomes of herpes viruses were not known.

**Fig 1:**
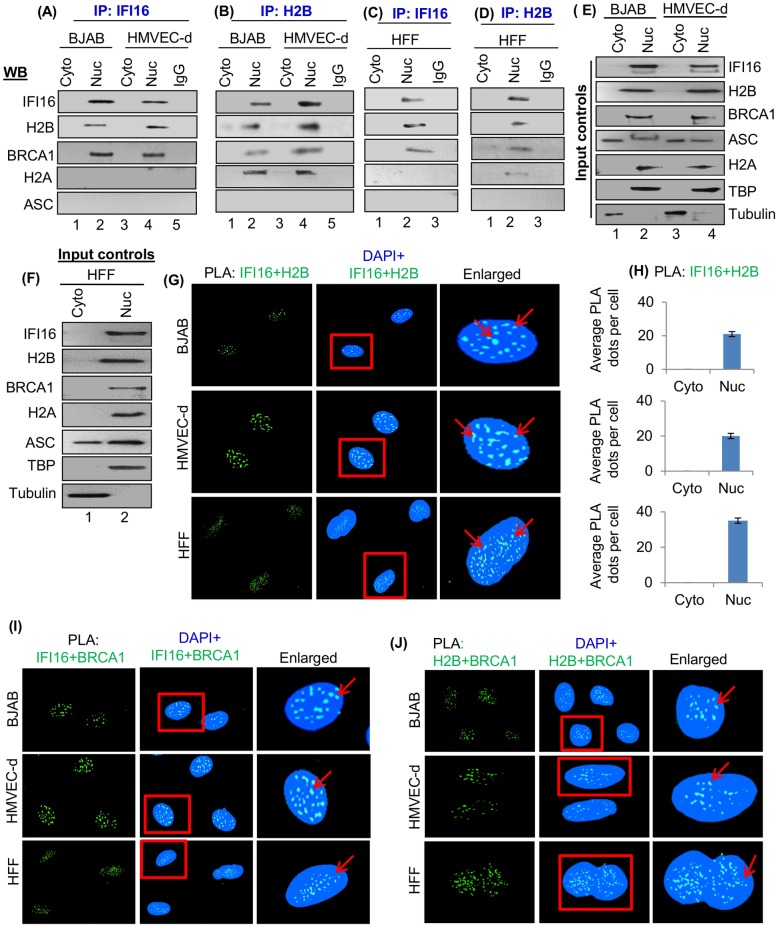
Demonstration of IFI16 interactions with H2B in the nuclear fraction of uninfected cells. (A-D) Cytoplasmic and nuclear fractions from uninfected BJAB, HMVEC-d and HFF cells were immunoprecipitated (IP-ed) using anti-IFI16 and anti-H2B antibodies and immunoblotted for IFI16, H2B, BRCA1, H2A and ASC. The nuclear fraction from HMVEC-d cells was IP-ed with control IgG antibody for specificity control. (E and F) Uninfected cytoplasmic and nuclear fractions were western blotted with the above antibodies for equal inputs. TBP and Tubulin were used to monitor the purity of the nuclear and cytoplasmic fractions, respectively. (G and H) Uninfected BJAB, HMVEC-d and HFF cells were subjected to PLA reactions using anti-IFI16 and H2B antibodies as described in Materials and Methods. Boxed areas were enlarged and interactions of IFI16 with H2B are indicated by the red arrows. Bar diagrams represent quantitation of the average number of dots per cell in the cytoplasm and nucleus of uninfected cells. (I and J) The above cells were also tested by PLA using anti-IFI16, BRCA1 and H2B antibodies. Boxed areas were enlarged and localization of IFI16 with BRCA1 and H2B with BRCA1 are indicated by the red arrows. Nuclei were stained with DAPI.

To validate the MS data, cytoplasmic and nuclear fractions from uninfected human B (BJAB-lymphoma), endothelial (HMVEC-d) and fibroblast (HFF) cells were IP-ed with anti-IFI16 and H2B antibodies and western blotted for various proteins. TATA-binding protein (TBP) and tubulin showed the purity of nuclear and cytoplasmic fractions, respectively, and the expression of IFI16, H2B, BRCA1, H2A and ASC proteins are shown by the input controls in Fig 1E and 1F. Results revealed the interaction of IFI16 and H2B only in the nuclear fractions in all three cell types examined (Fig 1A–1D). We have previously demonstrated that IFI16 interacts with BRCA1 in the uninfected cell nucleus [1]. Interestingly, we also observed the interaction of H2B with BRCA1 only in the nuclear fractions of HMVEC-d, HFF and BJAB cells (Fig 1B and 1D). Interactions between IFI16 and BRCA1 and between H2B and H2A were observed only in the nuclear fractions which served as positive controls (Fig 1A–1D). In contrast, no apparent interactions of IFI16 with ASC and H2A or H2B with ASC were observed (Fig 1A–1D).

### Proximity ligation assay (PLA) confirms IFI16-H2B and H2B-BRCA1 interactions

We performed PLA in uninfected BJAB, HMVEC-d and HFF cells as PLA detects an endogenous individual protein or interaction of two proteins based on the principle that if two epitopes or proteins are within the proximity of 40 nm or below, the PLA oligo probes linked to two secondary antibodies bound to primary antibody-antigen complexes can be amplified to give a PLA signal visualized as a fluorescent dot. PLA results demonstrated the close association (interaction) of IFI16 with H2B and BRCA1 and between H2B and BRCA1 only in the nucleus of uninfected cells (Fig 1G–1J, red arrows). H2B-H2A association and IFI16-ASC association were used as PLA controls, and quantitation of the average dots per cell are shown in S1A–S1D Fig. We did not observe any association between IFI16 and H2A and between H2B and ASC (S1E and S1F Fig). Primary, secondary or IgG control antibodies used to ascertain the specificity of PLA reactions did not show any amplified dots (S2A Fig). In addition, IFA results also supported the association of IFI16 and H2B only in the nucleus of uninfected BJAB and HMVEC-d cells (S2B Fig). These results validated our MS data and demonstrated the association of IFI16 with H2B in the nucleus of uninfected cells.

### H2B-IFI16 and H2B-BRCA1 complexes redistribute to the cytoplasm of HMVEC-d cells during *de novo* KSHV infection

**Fig 2:**
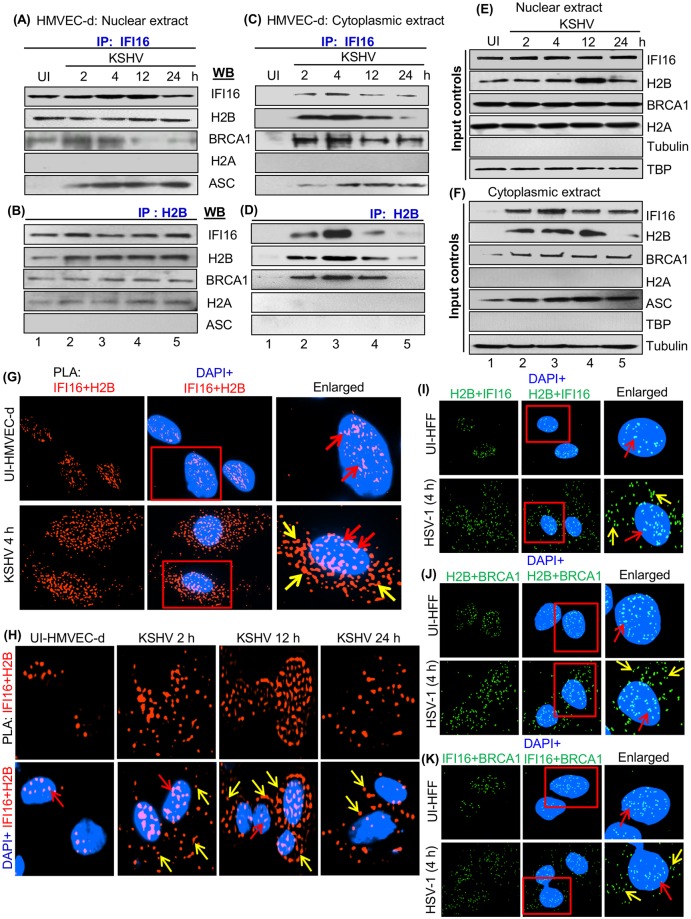
Immunoprecipitation and PLA analysis demonstrating the interaction and redistribution of IFI16 and H2B during KSHV *de novo* infection in HMVEC-d cells. (A-D) Nuclear and cytoplasmic fractions from uninfected and KSHV (30 DNA copies/cell) infected HMVEC-d cells at various time points (2, 4, 12, 24 h p.i.) were IP-ed using anti-IFI16 and anti-H2B antibodies and immunoblotted for IFI16, H2B, BRCA1, H2A and ASC. (E and F) Nuclear and cytoplasmic fractions were analyzed by WB for input controls, while tubulin and TBP WB showed the purity of cytoplasmic and nuclear fractions, respectively. (G and H) PLA analysis for the interaction of IFI16 with H2B at various time points of KSHV post-infection in HMVEC-d cells. Uninfected and KSHV (30 DNA copies/cell) infected for 4 h (G) and for 2, 12 and 24 h p.i. (H) HMVEC-d cells were subjected to PLA reactions using anti-IFI16 (mouse) and anti-H2B (rabbit) antibodies. Boxed areas were enlarged and the nuclear and cytoplasmic localization of IFI16-H2B are indicated by red and yellow arrows, respectively. (I-K) PLA analysis for the association and redistribution of H2B-IFI16 during HSV-1 *de novo* infection. Uninfected and HSV-1 infected (1 pfu/cell) (4 h) HFF cells were subjected to PLA reactions using anti-H2B, anti-IFI16, and anti-BRCA1 antibodies. Boxed areas were enlarged and H2B-IFI16, H2B-BRCA1 and IFI16-BRCA1 localization in the nucleus and cytoplasm are indicated by red and yellow arrows, respectively. Nuclei were stained with DAPI. Magnification: 40X.

Interaction of IFI16 with ASC and procaspase-1 along with BRCA1 results in inflammasome responses during KSHV, EBV and HSV-1 *de novo* infection and in cells carrying latent KSHV and EBV genomes [1–6]. Since we observed IFI16-H2B interaction in the nucleus of uninfected cells, we determined whether this interaction has any role in the inflammasome and IFN-β responses. Nuclear and cytoplasmic fractions from uninfected and KSHV infected HMVEC-d cells were IP-ed with anti-IFI16 or anti-H2B antibodies. Immunoblot results revealed an IFI16 and H2B interaction in the nuclear extracts of both uninfected and infected cells (Fig 2A and 2B, lanes 1–5). Interestingly, the IFI16-H2B interaction was also observed in the cytoplasmic extracts of cells infected with KSHV for 2, 4, and 12 h, which was reduced at 24 h p.i. (Fig 2C and 2D, lanes 2–5). In contrast, we observed little or no interaction in the cytoplasm of uninfected cells (Fig 2C and 2D, lane 1). IFI16 interacted with BRCA1 and ASC but not with H2A, and similarly, H2B interacted with BRCA1 and H2A but not with ASC in the nuclear extracts (Fig 2A and 2B). Moreover, in the cytoplasmic extracts of infected cells and not in uninfected cells, we observed the interactions of IFI16 with ASC and BRCA1 but not with H2A. Similarly, H2B was IP-ed with BRCA1 but not with H2A and ASC in the cytoplasm (Fig 2C and 2D). The expression levels of the proteins in nuclear and cytoplasmic extracts demonstrated that KSHV infection did not alter their levels (Fig 2E and 2F, input controls).

To confirm IFI16-H2B complex association and redistribution, uninfected and KSHV (4 h) infected cells were subjected to PLA using anti-IFI16 and anti-H2B antibodies. We observed substantial IFI16-H2B PLA spots in the nucleus of uninfected and KSHV infected cells (Fig 2G, red arrows; S2C Fig and Fig 2H). In contrast, significant levels of IFI16-H2B complex PLA spots were observed only in the cytoplasm of infected cells (Fig 2G, yellow arrows and S2C Fig). When PLA and IFA were performed to assess the IFI16-H2B complex at various times of infection, we observed increased association of IFI16 and H2B in the cytoplasm of infected cells from 2 to 12 h p.i. which was reduced at 24 h p.i. (Fig 2H, yellow arrows and S2D and S2E Fig).

In cells infected with vaccinia virus replicating its dsDNA in the cytoplasm of infected cells, we have demonstrated the activation of the cytoplasmic AIM2-ASC inflammasome and not the IFI16-ASC inflammasome, as well as no significant increase in the IFI16-BRCA1 interactions and the absence of IFI16-BRCA1 in the cytoplasm [1,2]. When we examined the H2B-IFI16 in vaccinia virus infected cells (5 pfu/cell; 4h), we did not observe any significant redistribution of nuclear H2B-IFI16 or H2B into the cytoplasm (S2G and S2H Fig).

In addition, we did not observe any significant association of IFI16 and H2A by PLA (S2F Fig). As demonstrated before [1, 3, 5], PLA and IFA results showed increased IFI16-ASC association PLA spots in the cytoplasm of infected cells at 2, 12 and 24 h p.i. which served as positive controls (S3A, S3B and S3C Fig).

These results demonstrated that IFI16 and H2B, and H2B and BRCA1 associate in the nucleus of uninfected and KSHV infected cells which redistribute to the cytoplasm only after infection and suggest that the presence of nuclear KSHV DNA is necessary for the H2B-IFI16 and H2B-BRCA1 cytoplasmic localization.

### HSV-1 *de novo* infection in HFF cells induces the cytoplasmic distribution of H2B-IFI16 and H2B-BRCA1 complexes

To determine whether H2B-IFI16 and H2B-BRCA1 interactions and their cytoplasmic distributions observed during *de novo* KSHV infected cells (Fig 2A–2H) also occur during other herpesvirus infections, we examined these interactions during HSV-1 *de novo* infection. Uninfected and HSV-1 (KOS) infected HFF cells (1 pfu/cell; 4 h) were subjected to a PLA reaction using anti-H2B, IFI16 and BRCA1 antibodies. We selected the 4 h time point as we have shown that beyond 4 h, IFI16 is not detected in the infected HFF cells as it is targeted and degraded by HSV-1 immediate early E3 ligase ICP 0 protein [1, 4, 6].

We observed the associations of H2B with IFI16, H2B with BRCA1 and the associations of IFI16 with BRCA1 in the nucleus of uninfected and HSV-1 infected cells (Fig 2I, 2J and 2K; red arrows). In contrast, significant associations of H2B with IFI16, H2B with BRCA1 and IFI16 with BRCA1 were observed only in the cytoplasm of infected cells (Fig 2I, 2J and 2K; yellow arrows). As demonstrated by us before [1], PLA spots indicating the IFI16-BRCA1 association and their cytoplasmic distribution served as a positive control (Fig 2K). These observations demonstrated that similar to KSHV, HSV-1 infection and the presence of nuclear viral genome also induces the redistribution of H2B-IFI16 and H2B- BRCA1 complexes to the cytoplasm of infected cells.

### The H2B-IFI16 and H2B-BRCA1 complexes redistribute to the cytoplasm of cells latently infected with KSHV

**Fig 3:**
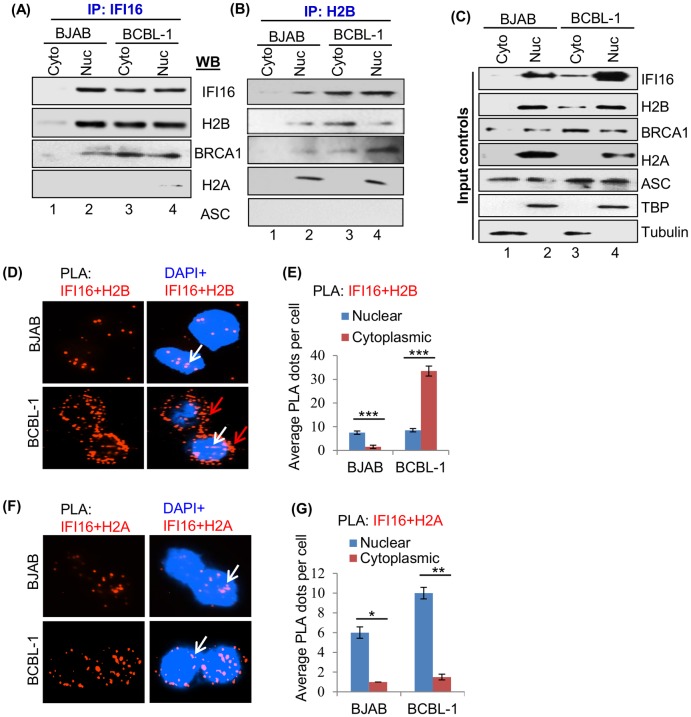
Demonstration of KSHV latent infection induced IFI16-H2B interaction and redistribution. (A and B) Cytoplasmic and nuclear extracts from BJAB and BCBL-1 cells were IP-ed using anti-IFI16 and anti-H2B antibodies, and immunoblotted with anti-IFI16, H2B, BRCA1, H2A and ASC antibodies. (C) Cytoplasmic and nuclear fractions were analyzed by WB for equal inputs, while tubulin and TBP confirmed the purity of cytoplasmic and nuclear extracts, respectively. (D-G) BJAB and BCBL-1 cells were tested by PLA using anti-IFI16 (mouse), anti-H2B (rabbit) and anti-H2A (rabbit) antibodies. Nuclear and cytoplasmic localization of IFI16-H2B and IFI16-H2A are indicated by white and red arrows, respectively (D and F). Nuclei were stained with DAPI. The average number of dots per cell in the nucleus and cytoplasm was quantitated and represented by bar graph (E and G). *, p<0.05; **, p<0.01; ***, p<0.001 nuclear vs. cytoplasmic dots.

We have shown constitutive IFI16-ASC-procaspase-1 inflammasome activation in association with BRCA1 in cells carrying multiple copies of latent KSHV episomal DNA and colocalization of IFI16 with the nuclear viral genomes [1, 3]. Since we observed IFI16 and H2B interaction in the nucleus of uninfected B (BJAB) cells (Fig 1A, 1B and 1G), we next determined their association and distribution during KSHV latent infection in B cells. Cytoplasmic and nuclear extracts from uninfected BJAB and KSHV latently infected BCBL-1 cells were IP-ed using anti-IFI16 and anti-H2B antibodies. Western blot analysis revealed the IFI16 and H2B interaction in the nucleus of BJAB and BCBL-1 cells, and interaction in the cytoplasm of BCBL-1 cells but not in BJAB cells (Fig 3A and 3B). As demonstrated before, IFI16 interacted with BRCA1 both in the nucleus and cytoplasm of BCBL-1 cells but only in the nucleus of BJAB cells. These, along with H2B interactions with H2A in the nucleus of BJAB and BCBL-1 cells, served as positive controls, while little or no observed association between IFI16 and H2A and no interaction between H2B and ASC served as negative controls (Fig 3A and 3B). Interestingly, we also observed the H2B interaction with BRCA1 in the cytoplasm of BCBL-1 cells but not in BJAB cells (Fig 3B). Expression levels of these proteins in these cells are shown in Fig 3C, input controls.

To further confirm the association and redistribution of IFI16-H2B, BJAB and BCBL-1 cells were tested by PLA using anti-IFI16 and anti-H2B antibodies. We observed considerable IFI16-H2B association PLA spots in the nucleus of BJAB and BCBL-1 cells (Fig 3D, white arrows). In contrast, significant redistribution of IFI16-H2B PLA spots was observed only in the cytoplasm of BCBL-1 cells (Fig 3D, red arrows and Fig 3E). Unlike the IP reactions in Fig 1, we also observed a moderate increase in IFI16 and H2A association in the nucleus of infected BCBL-1 cells compared to BJAB cells (Fig 3F and 3G). This could be due to the sensitivity of the PLA reaction detecting interactions that were either very weak or probably lost during IP-reactions. Nevertheless, these findings demonstrated that similar to *de novo* infection, latent KSHV infection induces the redistribution of the IFI16-H2B complex to the cytoplasm.

### The H2B-IFI16 complex redistributes to the cytoplasm of cells latently infected with EBV

Our previous studies have demonstrated the constitutive activation of IFI16 inflammasomes in association with BRCA1 in cells carrying multiple copies of latent EBV episomal DNA [1, 5], as well as the colocalization of nuclear IFI16 with the EBV genomes [5]. To determine whether H2B-IFI16 associate during EBV latent infection, EBV (-) BJAB, and EBV (+) LCL (latency III) and EBV (+) Akata (latency I) cells were subjected to IFA using anti-IFI16, H2B and BRCA1 antibodies. H2B-IFI16 colocalization was observed in the nucleus of uninfected BJAB cells (S4A Fig, white arrow). In contrast, significant colocalization of H2B-IFI16 was observed only in the cytoplasm of EBV+ LCL and Akata cells (S4A Fig; red arrows). Similar results were also observed for H2B-BRCA1 colocalization (S4B Fig) as well as for IFI16-BRCA1 colocalization used as a positive control [1] (S4C Fig).

Collectively, these observations suggest that latent EBV infection induces H2B-IFI16 and H2B-BRCA1 redistribution to the cytoplasm.

### KSHV *de novo* infection induces acetylation and translocation of H2B and IFI16

During *de novo* KSHV infection, viral genome recognition by nuclear IFI16 led into its acetylation by p300 and transport of acetylated IFI16 to the cytoplasm via Ran-GTPase [9]. Acetylation of H2B in the nucleus is one of the post-translational modifications essential for its function, such as interaction with DNA and proteins, transcription and chromatin remodeling [18, 19]. We next determined whether IFI16 associated H2B is also acetylated during infection to aid in cytoplasmic translocation. PLA reactions were performed using combinations of anti-acetyl lysine, H2B and IFI16 (rabbit or mouse) antibodies, and a non-toxic concentration of p300 competitive inhibitor C646 (1 μM) not affecting the viability of cells, viral entry or nuclear delivery of viral genome [9].

**Fig 4:**
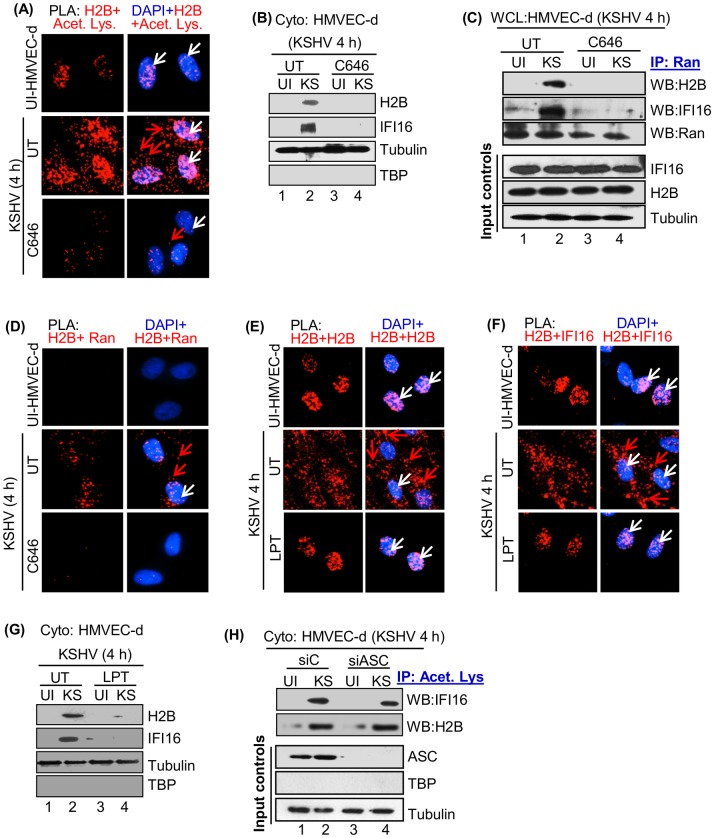
Acetylation of H2B and IFI16 during KSHV *de novo* infection. (A) Untreated HMVEC-d cells (UT) or cells preincubated with or without 1 μM of C646 for 2 h were infected with KSHV for 2 h, washed and incubated again for 2 h in the presence or absence of C646 and subjected to PLA analysis using anti-H2B (goat) and anti-acetyl lysine (rabbit) antibodies. White and red arrows indicate the nuclear and cytoplasmic localization of H2B-acetyl lysine, respectively. (B) Cytoplasmic fractions from uninfected and KSHV (KS) infected (4 h) cells treated with or without C646 (panel A) were western blotted with anti-IFI16 and anti-H2B antibodies. (C and D) Transport of H2B from the nucleus to the cytoplasm through Ran GTPase association during KSHV *de novo* infection. (C) Cells described in panel A were lysed in NETN-lysis buffer and whole cell lysates (WCL) were IP-ed using anti-Ran antibodies and immunoblotted for IFI16, H2B and Ran. The bottom panel shows the input controls for IFI16 and H2B. (D) Uninfected or KSHV infected cells treated with or without C646 from panel A were subjected to PLA reactions using anti-H2B (goat) and anti-Ran (rabbit) antibodies. Nuclear and cytoplasmic localization of H2B-Ran are indicated by white and red arrows, respectively. (E and F) Leptomycin B (LPT) blocks the nuclear export of IFI16-H2B. HMVEC-d cells preincubated in the presence or absence of LPT (50 nM) were uninfected or infected by KSHV for 2 h, washed, and incubated for 2 h in the presence or absence of LPT followed by PLA reactions using anti-H2B (goat or rabbit) and anti-IFI16 (mouse) antibodies. Nuclear and cytoplasmic localization of H2B-H2B (E) and H2B-IFI16 (F) are indicated by white and red arrows, respectively. (G) Cytoplasmic fractions from the above described cells (panels E and F) were immunoblotted with anti-H2B and anti-IFI16. (H) Effect of ASC on the acetylation of IFI16 and H2B. Cytoplasmic fractions from HMVEC-d cells electroporated with siC (control siRNA) and siASC for 48 h, infected with/without KSHV (4 h) were IP-ed with anti-acetyl lysine antibody and western blotted for IFI16 and H2B. The bottom panel shows the ASC knockdown efficiency.

As expected, acetylated H2B PLA spots were detected in the nucleus and not in the cytoplasm of uninfected cells (Fig 4A, top panel, white arrows). In contrast, we observed increased acetylated H2B PLA spots in the nucleus as well as in the cytoplasm of infected cells (Fig 4A, middle panel, white and red arrows), which were significantly reduced by C646 (Fig 4A, lower panel). As shown by us [9], PLA analysis for acetylated IFI16 revealed increased IFI16 acetylation and its localization in the cytoplasm of KSHV infected cells (positive control) which was abolished in the presence of C646 (S5A Fig). In addition, as before [9], distribution of IFI16 to the cytoplasm during KSHV infection was restricted to the nucleus in the presence of C646, demonstrating that only acetylated IFI16 redistributed to the cytoplasm (S5B Fig). To verify the PLA results (Fig 4A), cytoplasmic fractions from uninfected and KSHV infected (4 h) cells untreated (UT) or treated with C646 were tested with anti-H2B and anti-IFI16 antibodies (Fig 4B). Cytoplasmic H2B and IFI16 were detected only in the infected cells which was abolished by C646 (Fig 4B, lanes 2 and 4). Together, these findings demonstrated that similar to IFI16, KSHV infection induced the acetylation of H2B and its distribution to the cytoplasm.

### Ran-GTPase is critical for transport of H2B from nucleus to cytoplasm during *de novo* KSHV infection

Since we have shown that Ran-GTP assists the transport of acetylated IFI16 from the nucleus to the cytoplasm [9], we examined whether Ran-GTP is also involved in the transport of acetylated H2B. Whole cell lysate (WCL) from uninfected and KSHV infected cells (4 h) treated with or without C646 was IP-ed using anti-Ran-GTPase antibody. Immunoblot analysis of Ran-IFI16 association demonstrated that Ran was not associated with IFI16 in uninfected cells while a prominent association of Ran with IFI16 was observed in infected cells which was reduced by C646 (Fig 4C, second panel, lanes 1–4). Similarly, Ran was not associated with H2B in uninfected cells, and in contrast, a substantial association of Ran with H2B was detected in infected cells which was abolished by C646 (Fig 4C, top panel, lanes 1–4). Furthermore, PLA analysis demonstrated increased Ran-H2B association PLA spots only in the infected cell nucleus and cytoplasm (Fig 4D, white and red arrows, respectively) which was blocked by C646 (Fig 4D). These results demonstrated that KSHV infection induces the increased H2B acetylation which is crucial for H2B-Ran association followed by transportation to the cytoplasm of infected cells.

### Leptomycin B (LPT) abolishes transportation of the IFI16-H2B complex from nucleus to cytoplasm

After their translation in the cytoplasm, IFI16 and H2B translocate to the nucleus via their NLS domains [20, 21]. IFI16 redistribution to the cytoplasm during KSHV infection was inhibited by LPT [9]. To determine whether the H2B protein detected in the cytoplasm during KSHV infection represents newly synthesized molecules or redistributed from the nucleus, HMVEC-d cells pre-incubated with or without LPT (50 nM) were infected with KSHV for 4 h or uninfected in the presence or absence of LPT. The concentration of LPT used did not show any toxic effect on HMVEC-d cells nor on KSHV entry into the cells and gene expression [9]. PLA analysis revealed the redistribution of H2B (Fig 4E, red arrow) and H2B-IFI16 complex (Fig 4F, red arrow) to the cytoplasm only in the infected cells which was significantly blocked by LPT and restricted to the nucleus (Fig 4E and 4F, white arrows). In addition, western blot analysis using anti-H2B and anti-IFI16 antibodies with cytoplasmic fractions corroborated the finding that LPT blocked the redistribution of H2B and IFI16 to the cytoplasm (Fig 4G). These results demonstrated that the H2B-IFI16 complex detected in the cytoplasm originated from the nucleus of infected cells.

### ASC does not affect H2B acetylation during KSHV *de novo* infection

Although ASC knockdown abolished the IFI16-inflammasome, we could still detect reduced levels of IFI16 in the cytoplasm of KSHV infected HMVEC-d cells [9]. Hence, we sought to determine whether this IFI16 represented IFI16-H2B complex. Cytoplasmic extracts from HMVEC-d cells electroporated with siC (control siRNA) and siASC and infected with KSHV for 4 h or left uninfected were IP-ed with anti-acetyl lysine antibody. The knockdown efficiency of ASC is shown in Fig 4H, third panel. As seen in the PLA results (Fig 4A, top panel), a basal level of H2B acetylation was observed in uninfected cells (Fig 4H, panel 2, lane 1). An increase in acetylated cytoplasmic H2B levels in infected cells (Fig 4H, panel 2, lane 2) was observed, which was not affected by the absence of ASC (Fig 4H, panel 2, lanes 2 and 4). Only a moderate decrease in the level of cytoplasmic acetylated IFI16 was observed by ASC knockdown in infected cells (Fig 4H, panel 1, lanes 2 and 4).

Taken together, these observations suggest that: a) KSHV *de novo* infection increases nuclear H2B acetylation which is subsequently transported to the cytoplasm via Ran-GTP; b) ASC does not play any role in H2B acetylation and its cytoplasmic transportation; and c) IFI16-H2B is an independent complex distinct from the IFI16-ASC-procaspase-1-inflammasome complex formed during KSHV infection.

### KSHV *de novo* infection induces the interactions of H2B-STING and IFI16-STING in the cytoplasm

After stimulation, STING, an ER membrane protein activates TBK1, which in turn phosphorylates and activates IRF3, and pIRF3 translocates into the nucleus to initiate IFN-β gene transcription [12]. With our observations of increased acetylation of H2B and IFI16, H2B-IFI16 translocation to the cytoplasm independent of the IFI16-ASC inflammasome complex, and the induction of IFN-β in the absence of ASC in herpesvirus infected cells [6] together with the reported role of extra-chromosomal cytoplasmic H2B in interferon induction [17], we next determined whether H2B along with IFI16 plays any role in STING activation to induce IFN-β.

**Fig 5:**
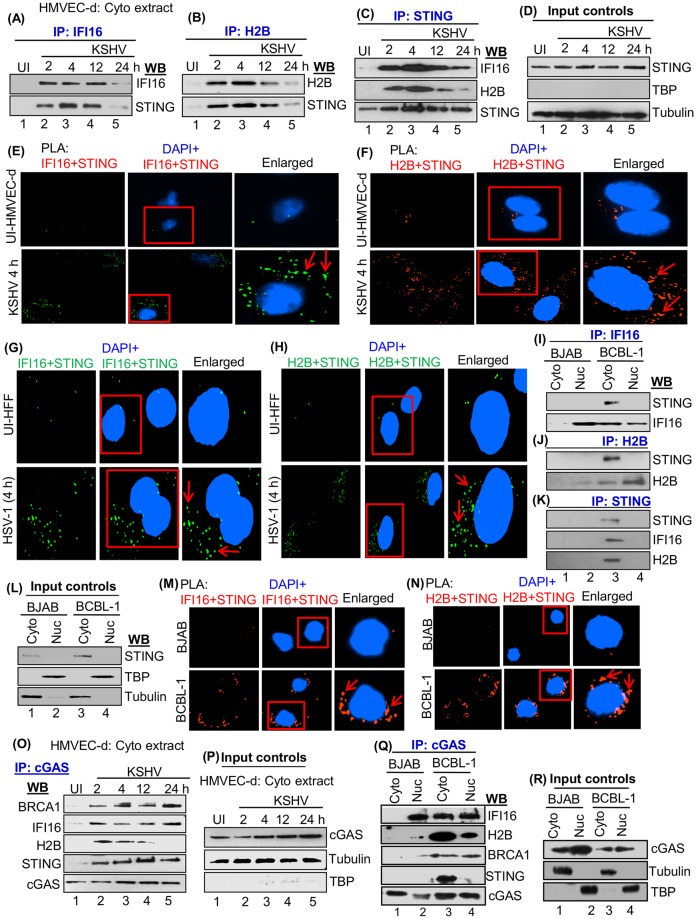
Demonstration of IFI16 and H2B association with STING during KSHV and HSV-1 *de novo* infection. (A, B and C) Cytoplasmic fractions from uninfected and KSHV infected HMVEC-d cells at 2, 4, 12, and 24 h p.i. were taken from Fig 2F and IP-ed using anti-IFI16, H2B and STING antibodies and western blotted for IFI16, H2B, and STING. (D) The cytoplasmic fraction was immunoblotted for STING for input control and tubulin and TBP for purity of the fraction. (E and F) Uninfected and KSHV infected (4 h) HMVEC-d were tested by PLA using anti-IFI16, H2B and STING antibodies. Boxed areas were enlarged. Red arrows represent the localization of IFI16-STING and H2B-STING in the cytoplasm. (G and H) PLA analysis demonstrating the association of IFI16-STING and H2B-STING during HSV-1 *de novo* infection. Uninfected and HSV-1 infected (1 pfu/cell) (4 h) HFF cells were subjected to PLA reactions. Boxed areas were enlarged and localization of IFI16-STING and H2B-STING in the cytoplasm is represented by red arrows. Magnification: 40X. (I, J and K) IFI16 and H2B association with STING during KSHV latent infection. Cytoplasmic and nuclear extracts from BJAB and BCBL-1 cells shown under Fig 3C experiments were IP-ed using anti-IFI16, H2B and STING antibodies and western blotted for IFI16, H2B, and STING. (L) Equal input of STING analyzed by WB. (M and N) BJAB and BCBL-1 cells were tested by PLA reactions using anti-IFI16 (mouse), H2B (goat) and STING (rabbit) antibodies. Red arrows represent the localization of IFI16-STING and H2B-STING in the cytoplasm. Nuclei were stained with DAPI. Magnification: 40X. (O) Association of cGAS with IFI16, H2B, STING and BRCA1 during KSHV *de novo* infection. Cytoplasmic fractions from uninfected and KSHV infected HMVEC-d cells at 2, 4, 12, and 24 h p.i. were IP-ed using anti-cGAS antibody and western blotted for IFI16, H2B, STING, BRCA1 and cGAS. (P) Input control for cGAS in the cytoplasmic fraction by WB. (Q and R) Association of cGAS with IFI16, H2B, STING and BRCA1 during KSHV latent infection. (Q) Cytoplasmic and nuclear fractions from BJAB and BCBL-1 cells were IP-ed with anti-cGAS antibody and western blotted for IFI16, H2B, STING, BRCA1 and cGAS. (R) Western blot showing input control for cGAS in cytoplasmic and nuclear fractions.

Cytoplasmic fractions from uninfected and KSHV infected HMVEC-d cells (2, 4, 12, 24 h p.i.) described in Fig 2F experiments were IP-ed with anti-IFI16, H2B or STING antibodies, and the results demonstrated the interactions of IFI16, H2B and STING (Fig 5A, 5B and 5C). Interaction of IFI16 with STING was observed from 2 to 24 h p.i., while the interaction of H2B with STING was reduced at 24 h p.i. (Fig 5A, 5B and 5C). In contrast, we did not detect any such interactions in uninfected cells (Fig 5A, 5B and 5C, lane 1). STING expression was not affected during KSHV infection (Fig 5D, input controls). To further confirm the interaction of IFI16, H2B and STING, uninfected and KSHV (4 h) infected cells were tested by PLA using anti-IFI16, H2B and STING antibodies and quantitated (S5C and S5D Fig). We observed substantial association between IFI16 and STING as well as between H2B and STING only in the cytoplasm of infected cells (Fig 5E and 5F, red arrows), demonstrating that these associations are induced by KSHV infection.

### Infection with UV-inactivated KSHV induces the interactions of H2B-STING and IFI16-STING in the cytoplasm

UV light treatment of KSHV abolishes its ability to express its genome. This process, however, does not affect the envelope and capsid of the virion, creating a virus that is still capable of entry into the virus and delivering the viral genome into the nucleus [2]. Previously, we have shown that the presence of nuclear viral genome but not viral gene expression is enough to induce the IFI16-ASC-procaspase-1 inflammasome activation in the nucleus and translocation into the cytoplasm [2]. Hence, we determined whether KSHV-induced H2B-STING and IFI16-STING is dependent on the presence of viral genome and/or viral gene expression. HMVEC-d cells were infected with 30 DNA copies/cell of UV-KSHV or live-KSHV and PLA reactions were performed for 2, 4 and 24 h p.i. using anti-H2B, IFI16 and STING antibodies. No H2B-STING or IFI16-STING interactions were observed in the uninfected cells (S5E–S5H Fig, top panel). In contrast, we observed significant H2B-STING and IFI16-STING interactions in UV-KSHV infected cells which were similar to live-KSHV infected cells, and we did not observe any significant change in the above associations between UV-KSHV and live KSHV infected cells (S5E, S5F, S5G and S5H Fig). Taken together, these observations suggest that viral DNA sensing in the nucleus induce the translocations of H2B and IFI16 to the cytoplasm and their interactions with STING and viral gene expression is not required.

### HSV-1 *de novo* infection induces the interactions of H2B-STING and IFI16-STING in the cytoplasm

To determine whether H2B-STING and IFI16-STING interactions in the cytoplasm observed in KSHV infected cells also occur during HSV-1 infection, uninfected or HSV-1 (KOS) (1 pfu/cell) infected HFF cells were subjected to PLA reactions using anti-STING, H2B and IFI16 antibodies. We observed substantial levels of H2B-STING and IFI16-STING PLA interaction spots in the cytoplasm of infected cells (Fig 5G and 5H, lower panels red arrow), which is in contrast to uninfected cells showing no or only a few spots of such interactions (Fig 5G and 5H, top panels). These results demonstrate that HSV-1 infection induces the cytoplasmic associations of H2B-STING and IFI16-STING early during infection.

### KSHV latent infection induces the interactions of H2B-STING and IFI16-STING in the cytoplasm

Cytoplasmic and nuclear fractions from KSHV (-) BJAB and KSHV (+) BCBL-1 cells described in Fig 3C experiments were IP-ed using anti-IFI16, H2B or STING antibodies. Tubulin and TBP western blots demonstrated the purity of the cytoplasmic and nuclear fractions, respectively (Fig 5L). Western blot analysis revealed the interactions between IFI16 and STING as well as between H2B and STING only in the BCBL-1 cytoplasmic fractions (Fig 5I, 5J and 5K, lane 3). The expression of STING in BJAB and BCBL-1 cells is shown in the input controls (Fig 5L).

PLA results demonstrated the IFI16-STING and H2B-STING interactions only in the cytoplasm and not in the nucleus of BCBL-1 cells (Fig 5M and 5N, red arrows, and S5I Fig). Specificities of these reactions are shown by the absence of PLA dots in single species primary anti-H2B or anti-IFI16 antibody reactions (S6A and S6B Fig). To rule out the role of ASC and H2A in the H2B-IFI16-STING interaction, WCL from uninfected and KSHV-infected (4 h) HMVEC-d cells were IP-ed with anti-STING or anti-ASC antibodies. Western blots showed no association of STING with H2A and between ASC and STING (S6C Fig) which was further confirmed by PLA analysis (S6D and S6E Fig).

Collectively, these results demonstrated the association of STING with IFI16 and H2B but not with ASC in the cytoplasm during KSHV *de novo* and latent infection.

### KSHV *de novo* and latent infection induces the interactions of cGAS with IFI16, H2B, BRCA1 and STING

cGAS (cGAMP-Synthase) is a cytosolic DNA sensor [12, 22], and studies suggest that IFI16, BRCA1 and cGAS are essential for IFN-β induction during HSV-1 infection of HFF cells [1, 10]. Hence, we evaluated whether cGAS is part of the IFI16-H2B complex. Cytoplasmic fractions from uninfected and infected HMVEC-d cells were IP-ed with anti-cGAS antibodies. We observed the interactions of cGAS with IFI16, BRCA1 and STING in the cytoplasmic extracts of KSHV infected cells at 2, 4, 12 and 24 h p.i. Interestingly, cGAS interacted with H2B at 2, 4, and 12 h p.i. with KSHV which was reduced at 24 h p.i. (Fig 5O, lanes 2–5). In contrast, very little or no association of the above proteins was observed in uninfected cell cytoplasm (Fig 5O, lane 1). Expression levels of cGAS remained unchanged in the cytoplasm of infected cells (Fig 5P). Input controls for BRCA1, IFI16, H2B and STING were similar as in Figs 2F and 5D. These results suggested that cGAS interacts with IFI16, H2B, BRCA1 and STING in the cytoplasm of KSHV infected HMVEC-d cells.

Next, we determined the interactions of cGAS with IFI16, H2B, BRCA1 and STING in cells latently infected with KSHV. Cytoplasmic and nuclear fractions from BJAB and BCBL-1 cells were IP-ed with anti-cGAS antibodies. Western blot analysis revealed that cGAS interacted with IFI16, H2B, and BRCA1 in the nucleus of BJAB and BCBL-1 cells (Fig 5Q, lane 2 and 4). cGAS also interacted with IFI16, H2B, BRCA1 and STING but only in the infected BCBL-1 cytoplasm, and in contrast, even though cGAS was detected in the cytoplasm of control BJAB cells (Fig 5R, lane 1), we did not observe any interaction with IFI16, H2B, BRCA1 and STING (Fig 5Q, lanes 1). The expression levels of cGAS is shown in the input controls (Fig 5R), and the levels of IFI16, H2B, BRCA1 and STING is shown in Figs 3C and 5L as input controls. These observations indicated that KSHV latent infection also induces the interactions of cGAS, IFI16, H2B, BRCA1 and STING in the cytoplasm of infected cells.

### Double PLA reactions demonstrate the associations of IFI16, H2B, BRCA1, cGAS and STING in the cytoplasm of KSHV infected cells

**Fig 6:**
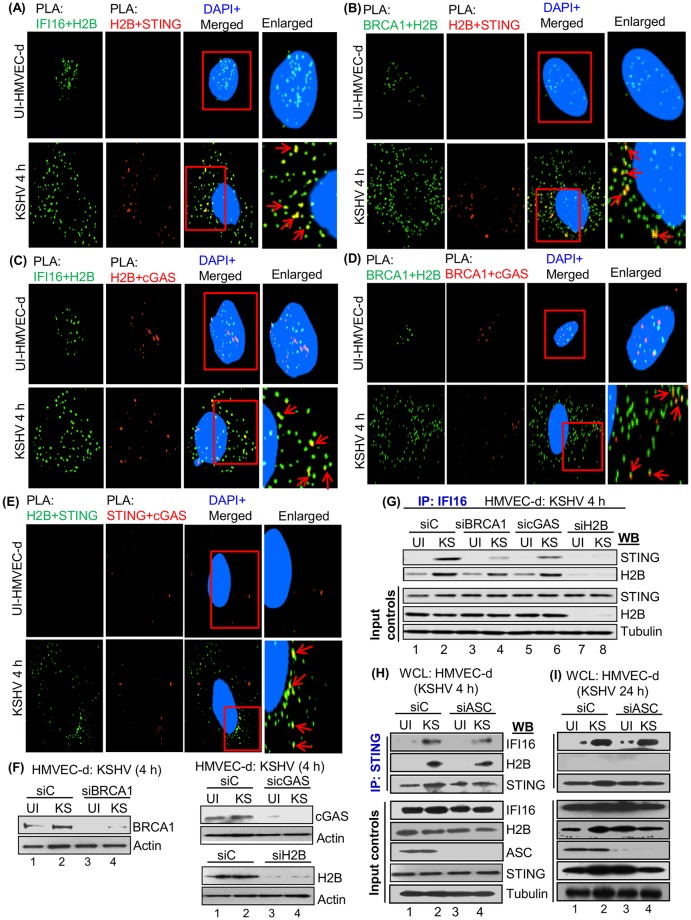
Association of IFI16, H2B, BRCA1, cGAS and STING during KSHV infection. (A) For a double PLA reaction, two independent reactions were performed. In the first PLA reactions, uninfected and KSHV (KS) infected (4 h) HMVEC-d cells were immunostained using mouse (ms) anti-IFI16 and goat (g) anti-H2B antibodies, and detected by DUOLink green detection agent. Cells were washed, blocked and subjected to the second PLA reactions using goat anti-H2B and rabbit (rb) anti-STING antibodies and visualized with red detection agents. Green and red dots indicate the localization of IFI16-H2B and H2B-STING, respectively. (B-E) Similarly, double PLA for different combinations were performed using BRCA1 (ms) + H2B (g) and H2B (g) + STING (rb); IFI16 (ms) + H2B (rb) and H2B (rb) + cGAS (g); BRCA1 (ms) + H2B (rb) and BRCA1 (ms) + cGAS (g); and H2B (ms) + STING (rb) and STING (rb) + cGAS (g). Red arrows indicate yellow dots representing the associations of three different proteins together such as IFI16-H2B-STING (A), BRCA1-H2B-STING (B), IFI16-H2B-cGAS (C), BRCA1-H2B-cGAS (D) and H2B-STING-cGAS (E) in the cytoplasm of KSHV infected cells. Nuclei were stained with DAPI. (F and G) Effect of BRCA1, cGAS and H2B on the interaction of IFI16-STING during KSHV *de novo* infection. (F) WCL from HMVEC-d cells electroporated with siC, siBRCA1, sicGAS and siH2B for 48 h then infected or uninfected with KSHV for 4 h were western blotted with anti-BRCA1, cGAS and H2B antibodies. (G) The WCL lysates from panel F were IP-ed with anti-IFI16 antibody and immunoblotted for STING and H2B. The bottom panels showed H2B knockdown efficiency and STING as an input control. (H and I) Role of ASC in the association of STING with IFI16 and H2B during KSHV *de novo* infection. WCL from HMVEC-d cells electroporated with siC or siASC for 48 h followed by with and without KSHV infections (4 and 24 h) were IP-ed with anti-STING antibody and immunoblotted for IFI16, H2B and STING. The bottom panels show the knockdown efficiency of ASC along with equal inputs of IFI16, H2B and STING by WB.

Interactions of IFI16 and H2B with STING in the cytoplasm during KSHV infection prompted us to determine whether these proteins also interact with BRCA1 and cGAS in the cytoplasm of infected cells, and whether they form macromolecular complexes. For this, uninfected and infected HMVEC-d cells (4 h) were subjected to double sequential PLA reactions with initial reactions for a) IFI16 and H2B (Fig 6A and 6C, green spots), BRCA1+H2B (Fig 6B and 6D, green spots), and H2B+STING (Fig 6E, green spot), respectively, followed by b) second reaction indicated by red spots for H2B+STING (Fig 6A and 6B), H2B+cGAS (Fig 6C), BRCA1+cGAS (Fig 6D) and STING+cGAS (Fig 6E), respectively. We observed the following interesting results:

1. Similar to IFI16+BRCA1 (Fig 1I), IFI16+H2B and BRCA1+H2B interactions were localized only to the nucleus of uninfected cells. In contrast, substantial levels of IFI16+H2B and BRCA1+H2B PLA spots were detected in the cytoplasm of infected cells (Fig 6A–6D), and H2B+STING was detected only in the cytoplasm of infected cells (Fig 6A and 6B). This demonstrated that KSHV infection induced the cytoplasmic redistribution of IFI16+H2B and BRCA1+H2B, and H2B interactions with STING. Furthermore, colocalization of H2B+STING PLA spots with IFI16+H2B and BRCA1+H2B spots (Fig 6A and 6B, yellow spots and red arrows) suggested that the macromolecular complex formed between IFI16+H2B+BRCA1 is in close proximity with STING in the cytoplasm after infection.
2. Very few H2B+cGAS and BRCA1+cGAS PLA spots were detected in the nucleus of uninfected cells (Fig 6C and 6D) and they did not colocalize with the few IFI16+H2B and BRCA1+H2B spots, respectively, indicating that these could be independent complexes in the nucleus (Fig 6C and 6D). However, few H2B+cGAS and BRCA1+cGAS red spots were detected in the cytoplasm of infected cells and the majority of these colocalized with IFI16+H2B and BRCA1+H2B spots, respectively (Fig 6C and 6D, yellow spots and red arrows). These results suggested that IFI16+H2B+cGAS and BRCA1+H2B+cGAS are in close proximity in the cytoplasm after infection.
3. H2B+STING and STING+cGAS spots were not detected in the uninfected cells (Fig 6E), indicating that they are not associated under physiological conditions. In contrast, a substantial number of H2B+STING interacting spots as well as a few STING+cGAS spots were detected in the cytoplasm of infected cells and most of the STING+cGAS spots colocalized with H2B+STING spots (Fig 6E, yellow spots and red arrows). This suggested that H2B+STING+cGAS are in close proximity in the cytoplasm and associate only after KSHV infection.

Taken together, these results demonstrated that during KSHV infection, nuclear H2B associated with IFI16 and BRCA1, translocates to the cytoplasm and associates with cGAS-STING. These results further validated the IP-reactions in Fig 5.

### H2B, BRCA1 and cGAS play a role in the IFI16-STING interaction during KSHV *de novo* infection

Our recent studies demonstrated that BRCA1 knockdown impaired not only genome recognition by IFI16 but also the cytoplasmic IFI16-STING mediated IFN-β response during *de novo* KSHV and HSV-1 infection [1]. Previous studies also suggested that cGAS induced STING-dependent activation of IRF-3 signaling cascades [12, 22]. Since BRCA1 and cGAS have been shown to be involved in KSHV and HSV-1 IFN-β responses, we investigated whether H2B has any role in IFI16-STING mediated signaling.

HMVEC-d cells electroporated with siC, siBRCA1, sicGAS and siH2B were uninfected or infected with KSHV for 4 h, and whole cell lysates were IP-ed with anti-IFI16 antibodies. We observed efficient knockdown of these proteins (Fig 6F). In siC KSHV infected cells, STING was IP-ed with IFI16 which was abolished by H2B knockdown (Fig 6G, compare lanes 1 and 2 with 7 and 8). Similarly, little or no STING was IP-ed with IFI16 in BRCA1 knockdown infected cells (Fig 6G, compare lanes 1 and 2 with lanes 3 and 4), while cGAS knockdown reduced (~50%) the levels of STING associated with IFI16 (Fig 6G, compare lanes 1 and 2 with lanes 5 and 6). In addition, BRCA1 and cGAS knockdown also hampered the interaction of IFI16-H2B by ~40% and ~25%, respectively (Fig 6G). The bottom input panels of Fig 6G show the efficiency of H2B knockdown, the absence of off-target effects as well as STING levels.

These results suggested a critical participation of H2B and BRCA1 in the cytoplasmic IFI16-STING interactions during *de novo* KSHV infection.

### Inflammasome component ASC does not affect the interaction of STING with IFI16 and H2B during KSHV *de novo* infection

Our studies show that in the absence of ASC, acetylated H2B and IFI16 are detected in the cytoplasm of infected cells (Fig 4H). To rule out the effect of ASC on the H2B-STING interaction, HMVEC-d cells electroporated with siC and siASC were infected with KSHV for 4 and 24 h, and uninfected and infected cell WCL were IP-ed using anti-STING antibodies. ASC knockdown efficiency and expression levels of IFI16, STING and H2B are shown in Fig 6H and 6I, bottom panels. The absence of ASC did not impact the interaction of H2B with STING or IFI16 with STING in cells infected with KSHV for 4 h (Fig 6H, top three panels). In contrast, although IFI16 IP-ed with STING, the interaction between H2B and STING was not detected at 24 h p.i. (Fig 6I, top three panels) which is similar to the observations in Fig 5B and 5C demonstrating the reduced H2B levels and H2B-IFI16 interaction in the cytoplasm at 24 h p.i. Targeting of H2B by factors (host and/or viral) could be a potential reason for such a reduced interaction with STING during *de novo* KSHV infection.

Nevertheless, these findings suggest that IFI16, H2B, BRCA1, cGAS and STING associate in the cytoplasm during KSHV *de novo* and latent infection that is independent of ASC.

### H2B is essential for IFN-β induction during KSHV *de novo* infection

**Fig 7:**
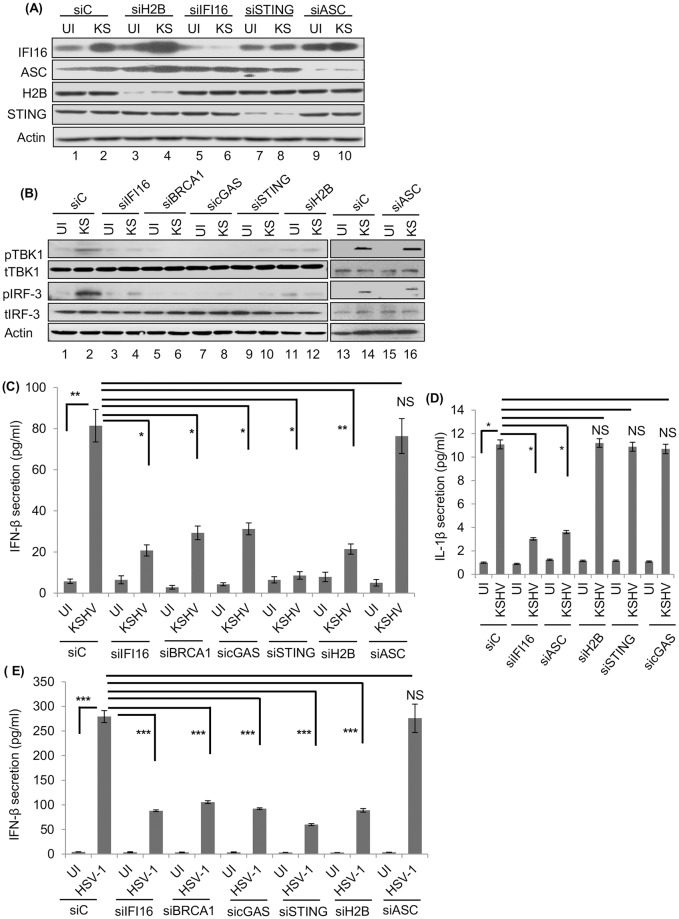
Effects of IFI16, H2B, BRCA1, cGAS, STING and ASC knockdown on IFN-β during KSHV and HSV-1 infection. (A) WCL from HMVEC-d cells electroporated with siC, siH2B, siIFI16, siSTING or siASC for 48 h followed by infection for 4 h with or without KSHV (KS) were western blotted with anti-IFI16, ASC, H2B and STING antibodies. (B) Phosphorylation of TBK1 and IRF3 in H2B, IFI16, BRCA1, cGAS, STING and ASC knockdown cells infected with KSHV. HMVEC-d cells electroporated with different siRNA (panel A and Fig 6F) for 48 h were followed by with or without KSHV infection (4 h), and immunoblotted using anti-pTBK1, tTBK1 (total), pIRF3 and tIRF3 antibodies. (C and D) Effect of IFI16, H2B, BRCA1, cGAS, STING and ASC knockdown on IFN-β and IL-1β secretion during KSHV *de novo* infection. (C) Cell culture supernatants from HMVEC-d cells electroporated with siC, siIFI16, siBRCA1, sicGAS, siSTING, siH2B or siASC for 48 h followed by infection (4 h) with or without KSHV (Fig 7A and Fig 6F), were collected and subjected to IFN-β ELISA. Results presented are means ± SD (* p<0.05, ** p<0.01 from siC vs. siIFI16, siBRCA1, sicGAS, siSTING, and siH2B with KSHV infection; NS: not significant). (D) The same supernatants from panel C were used to detect IL-1β secretion by ELISA. Results presented are means ± SD (* p<0.05, from siC vs. siIFI16, and siASC with KSHV infection; NS: not significant). (E) Effect of IFI16, H2B, BRCA1, cGAS, STING and ASC knockdown on IFN-β secretion during HSV-1 infection. Cell culture supernatants from HFF cells electroporated with siRNA (S6F Fig) for 48 h followed by with or without HSV-1 infection (4 h) were collected and IFN-β ELISA was performed. Results presented are means ± SD (*** p<0.001 from siC vs. siIFI16, siBRCA1, sicGAS, siSTING, or siH2B with HSV-1 infection; NS: not significant).

KSHV infection induces IFI16-mediated IL-1β and IFN-β secretion during *de novo* infection [1, 2, 9]. To determine the functional role of H2B, HMVEC-d cells electroporated with siC, siIFI16, siBRCA1, sicGAS, siSTING, siH2B or siASC and WCL from uninfected and KSHV (4 h) infected cells were western blotted. We observed efficient knockdown of these proteins (Figs 6F and 7A). To determine the levels of pTBK-1 and pIRF3 signaling molecules involved in IFN-β induction, WCL of these cells were immunoblotted with anti-pIRF3, tIRF3, pTBK-1 or tTBK-1 antibodies. We observed increased levels of pIRF3 and pTBK-1 in control siRNA (siC) KSHV infected cells (Fig 7B, lanes 1, 2, 13 and 14) which were significantly decreased in siIFI16, siBRCA1, sicGAS, siSTING, and siH2B infected cells (Fig 7B, lanes 3–12). In contrast, KSHV infection induced pIRF3 and pTBK-1 levels that were not affected in siASC cells (Fig 7B, lanes 15 and 16).

When supernatants from these cells were tested for IFN-β by ELISA, we observed increased IFN-β secretion (~80 pg/ml) in siC-KSHV infected cells (4 h) which was not affected in siASC knockdown cells (Fig 7C). In contrast, significant reduction in IFN-β secretion was observed in siH2B, siIFI16, siBRCA1, siSTING or sicGAS cells infected with KSHV (Fig 7C).

Taken together, these results demonstrated that H2B, IFI16, BRCA1, STING, and cGAS play roles in the activation of TBK, IRF3 and induction of IFN-β.

### H2B is not essential for IL-1β induction during KSHV *de novo* infection

When the same supernatants from Figs 6F and 7A experiments were tested for the secreted IL-1β levels, significant reduction was observed only in siIFI16 and siASC KSHV infected cells in comparison to siC infected cells (Fig 7D). In contrast, we did not observe any reduction in secreted IL-1β levels in sicGAS, siSTING and siH2B infected cells (Fig 7D) which clearly demonstrated that H2B, cGAS, and STING do not play any role in inflammasome activation and IL-1β secretion.

### H2B is essential for IFN-β induction during HSV-1 *de novo* infection in HFF cells

HSV-1 infection induced IFN-β secretion in primary HFF cells [6, 7]. To analyze the importance of H2B in IFN-β induction by HSV-1, HFF cells electroporated with siC, siIFI16, siBRCA1, sicGAS, siSTING, siH2B or siASC were infected with HSV-1 (4 h), and supernatants were tested by IFN-β ELISA. WB analysis of WCL from the above cells showed significant knockdown efficiency of these proteins (S6F Fig). We detected a significant level of IFN-β secretion (~280 pg/ml) in siC-HSV-1 infected cells which was unaffected in siASC-infected cells (Fig 7E). In contrast, a significant reduction in IFN-β secretion was observed in siH2B, siIFI16, siBRCA1, siSTING and sicGAS infected cells (Fig 7E).

Collectively, these findings demonstrated that similar to the role played by IFI16, H2B, BRCA1, STING and cGAS play a role in HSV-1 infection induced IFN-β production.

### Absence of H2B and IFI16 results in significant reduction of cGAMP production during KSHV *de novo* infection

**Fig 8:**
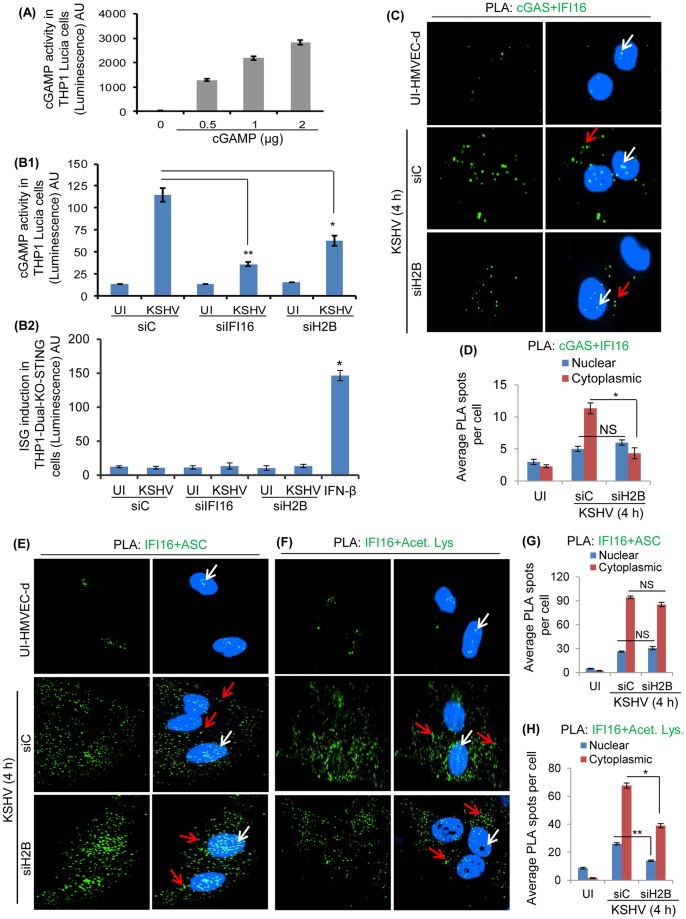
Effects of H2B knockdown on cGAMP production, IFI16-cGAS association, IFI16 acetylation, and IFI16-ASC cytoplasmic redistribution during KSHV *de novo* infection. (A) THP-1-Lucia ISG cells (1X10^5^) were treated with pure cGAMP (0–2 μg) to measure the activity of cGAMP used as a positive control. (B1 and B2) HMVEC-d cells electroporated with siC, siIFI16 and siH2B were infected with KSHV for 4 h. Cells were lysed, treated with benzonase for 30 min at 37°C, and heat inactivated at 95°C for 5 min. 10 μl of heat inactivated lysates were used to measure the cGAMP production using THP-1-Lucia ISG and THP-1-Dual KO-STING cells as described in the Materials and Methods. Recombinant human IFN-β at 1000 IU/ml was used as a positive control (B2). Results presented are means ± SD of three independent experiments. * p<0.05 and ** p<0.01 of siC vs. siIFI16 and siH2B with KSHV infection. (C, E and F) HMVEC-d cells electroporated with siC and siH2B followed by KSHV infection for 4 h were subjected to a PLA reaction using anti-IFI16, acetylated lysine, cGAS, and ASC antibodies. Nuclear and cytoplasmic spots are depicted by white and red arrows, respectively. (D, G and H) Average PLA spots of cGAS-IFI16, IFI16-ASC and IFI16-Acetyl lysine associations were quantitated and presented by bar graph. Results shown are means ± SD of three independent experiments. * p<0.05 and ** p<0.01 of siC vs. siH2B with KSHV infection; NS is not significant.

cGAS induction results in the production of cGAMP (cyclic GMP-AMP) which in turn activates STING resulting in pTBK-1, pIRF3 and interferon induction. To ascertain our findings that H2B and IFI16 are essential in IFN-β production through STING-mediated pathway during KSHV infection, we determined the level of cGAMP production. For this we used the THP1-Lucia cells expressing the secreted luciferase Lucia reporter gene under the control of an IRF-inducible promoter consisting of five IFN-stimulated response elements (ISRE). The activation of STING by cGAMP induces the IRF3 phosphorylation, pIRF3 translocation into the nucleus and activation of ISRE resulting in the secreted luciferase. Uninfected HMVEC-d cells electroporated with siC, siH2B and siIFI16 were infected with KSHV for 4 h, lysed, treated with benzonase, and heat inactivated at 95°C for 5 min. 10 μl of these lysates or varying amounts of purified cGAMP were added to 1X10^5^ THP-1-Lucia ISG cells. After overnight incubation, 10 μl culture supernatant was used in a luminescence assay as a measure of cGAMP produced during KSHV infection. A control luminescence assay measuring the cGAMP activity of pure cGAMP is shown in Fig 8A. We observed that KSHV infection induced a considerable level of cGAMP in siC HMVEC-d cells which was reduced significantly in the absence of IFI16 (~75%) and H2B (~60%) (Fig 8B1).

As a specificity control that the observed results shown in Fig 8B1 are via STING, we used the same lysates from KSHV infected cells with STING knockout (KO) THP-1-Dual KO-STING cells expressing secreted Lucia luciferase gene under the control of ISG54 (interferon-stimulated gene) ISRE which can be induced by STING-dependent IRF3 as well as STING-independent IRF9 that is inducible by IFN-α/β. When ISRE-induced Lucia luciferase activity was measured, the recombinant human IFN-β used as control increased the luciferase activity in THP-1-Dual KO-STING cells which demonstrated the activation by a STING-independent pathway (Fig 8B2). In contrast, we observed only negligible levels of luciferase activity in the supernatant of cells incubated with the lysates from siC KSHV and IFI16 and H2B knocked down infected cells (Fig 8B2). These results demonstrated that the luciferase activity seen in Fig 8B1 was through the cGAS-STING dependent pathway.

When these electroporated plus KSHV infected/uninfected cells (from Fig 8B1) were examined by PLA, compared to uninfected cells, a considerable number of IFI16-cGAS PLA spots were observed in the cytoplasm of siC KSHV infected cells (Fig 8C and 8D). In contrast, a significant reduction in the cytoplasmic IFI16-cGAS association was observed in H2B knockdown cells (Fig 8C and 8D). Together with the results shown in Figs 5, 6 and 7, these results demonstrate that H2B is essential for IFI16-cGAS association, cGAMP induction and IFN-β production during *de novo* KSHV infection.

### H2B knockdown effect on cytoplasmic distribution of IFI16-ASC and acetylated IFI16

To ascertain the results shown in Fig 7 that H2B doesn’t play a role in inflammasome activation and IL-1β secretion, uninfected HMVEC-d cells were electroporated with siC and siH2B and knockdown efficiencies were verified. These cells were infected with KSHV (30 DNA copies/cell) for 4 h and tested by PLA reactions using anti-IFI16 and anti-ASC antibodies. As reported by us before [1, 2], compared to uninfected cells, we observed a significant number of IFI16-ASC interacting PLA spots in the cytoplasm of siC-infected cells (Fig 8E and 8G). In addition, we did not observe any significant change in the association of IFI16 with ASC association in H2B knockdown cells (Fig 8E and 8G). These results, together with the negligible effect on the secreted IL-1β levels in siH2B infected cells (Fig 7D), demonstrated that IFI16-ASC inflammasome induction and their cytoplasmic translocation is independent of H2B.

When these cells were examined by PLA with anti-IFI16 and anti-acetylated antibodies, compared to uninfected cells, a substantial level of acetylated IFI16 PLA spots were observed in the cytoplasm of KSHV infected cells (Fig 8F and 8H). Knockdown of H2B resulted in ~30% reduction in the cytoplasmic acetylated IFI16 PLA spots (Fig 8F and 8H). These results suggested that the absence of H2B doesn’t affect IFI16-ASC association and cytoplasmic distribution but reduces the level of cytoplasmic acetylated IFI16 during KSHV *de novo* infection.

This reduction suggests that during KSHV infection, two distinct acetylated IFI16-H2B and IFI16-ASC complexes are formed in the nucleus and redistributed to the cytoplasm, and H2B knockdown results in the absence of acetylated IFI16-H2B in the cytoplasm.

### H2B is involved in nuclear KSHV and HSV-1 genome recognition by IFI16

**Fig 9:**
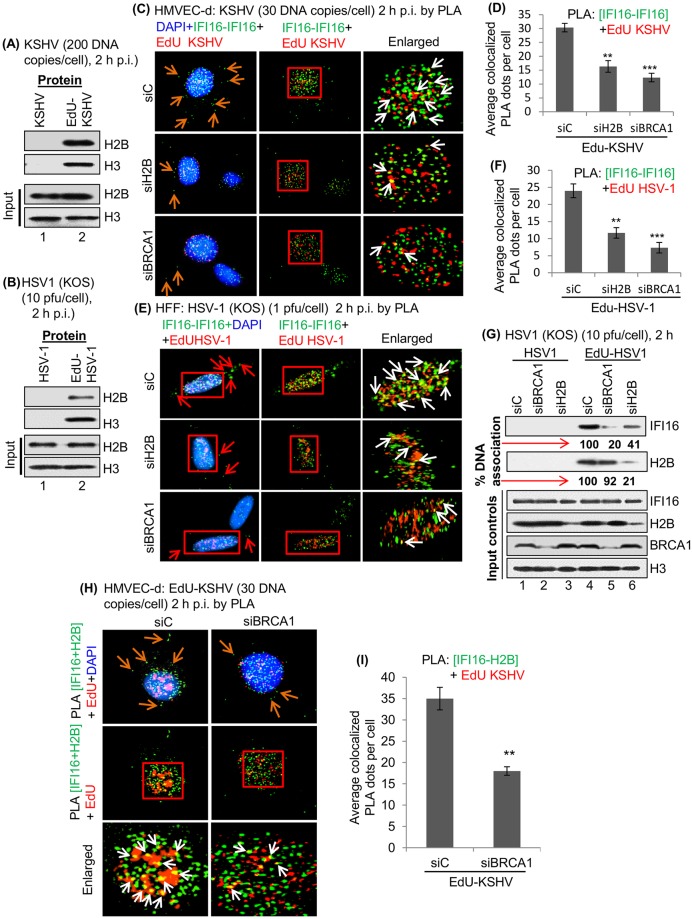
Detection of EdU labeled KSHV or HSV-1 genome associated host cell proteins by chromatin pull down during *de novo* infection. (A) HMVEC-d cells were infected by EdU labeled or unlabeled KSHV genome (200 DNA copies/cell) for 2 h and (B) HFF cells were infected by EdU labeled or unlabeled HSV-1 genome (10 PFU/cell) for 2 h. Protein-DNA cross-linking was performed, and biotin-TEG azide selectively linked to the reactive alkyne group of EdU containing DNA through a click reaction. DNA was sheared and short chromatin fragments captured on streptavidin beads. Pulled down proteins were analyzed by immunoblot using anti-H2B and H3 antibodies. (C-F) H2B is critical in KSHV or HSV-1 genome recognition by IFI16 during *de novo* infection. (C) HMVEC-d cells electroporated with siC, siH2B or siBRCA1 for 48 h were infected by EdU-labeled KSHV (30 DNA copies/cell) for 2 h and tested by PLA using mouse and rabbit anti-IFI16 (green dots). EdU labeled KSHV genome was detected by reaction with Alexa 594 labeled picolylazide (red dots). The presence of IFI16 and EdU labeled KSHV genome association (yellow color) was indicated by white arrows. Red arrows in the left panel indicate the distribution of IFI16 in the cytoplasm. (D) Bar graph showing the quantitation of IFI16-EdU-KSHV genome colocalization (yellow dots). ** p<0.01 and *** p<0.001 that represents siC vs. siH2B and siBRCA1 with EdU-KSHV infection. (E) HFF cells electroporated using siC, siH2B or siBRCA1 for 48 h were infected by EdU-labeled HSV-1 (KOS) with 1 PFU/cell for 2 h and tested for PLA as described in panel (C). The presence of IFI16 and EdU labeled HSV-1 genome association (yellow dots) is indicated by white arrows. Nuclei were stained with DAPI. (F) Average colocalized yellow dots per cell were quantitated and presented by bar graph. ** p<0.01 and *** p<0.001 that presents siC vs. siH2B and siBRCA1 with EdU-HSV-1 infection. (G) HFF cells electroporated with siC, siBRCA1 or siH2B for 48 h were infected by EdU-labeled or unlabeled HSV-1 genome (10 PFU/cell) for 2 h. Protein-DNA cross-linking was performed as described in panel B. Pulled down protein was analyzed by immunoblot using anti-H2B and anti-IFI16 antibodies. The bottom panel shows the input controls for IFI16, H2B, BRCA1 and H3. (H) Effect of BRCA1 on KSHV genome recognition by IFI16-H2B during *de novo* infection. HMVEC-d cells electroporated with siC or siBRCA1 for 48 h were infected by EdU-labeled KSHV (30 DNA copies/cell) for 2 h and tested by PLA using mouse anti-IFI16 and rabbit anti-H2B (green dots). EdU labeled KSHV genome was detected by red dots. Boxed areas were enlarged and the presence of IFI16-H2B and EdU labeled KSHV genome association (yellow color) was indicated by white arrows (bottom panel). Red arrows in the top panel indicate the redistribution of IFI16-H2B in the cytoplasm. (I) The average number of colocalized yellow PLA dots per cell as shown in Fig 9H was quantitated and presented in the bar graphs. **p<0.01 from siC vs. siBRCA1 with EdU KSHV infection.

We have reported that IFI16 recognizes the episomal KSHV, EBV and HSV-1 genomes in the nucleus of infected cells resulting in IFI16 mediated innate inflammasome and IFN-β responses [1, 2, 4–6]. Herpesvirus genomes delivered in the nucleus as linear, naked dsDNA with nicks and breaks undergo rapid circularization and chromatinization [23]. Since H2B is an essential component of chromatin structure, we first determined whether H2B associates with the KSHV genome in the nucleus. HMVEC-d cells were infected with unlabeled or EdU-genome labeled KSHV (200 copies/cell) for 2 h. A DNA mediated pull down assay was performed as described earlier [1] by first cross-linking protein-DNA and linking biotin-TEG azide to EdU-labeled viral DNA via a Click reaction. Following pull down by streptavidin, captured proteins were immunoblotted with anti-H2B and anti-H3 antibodies. Similarly, HSV-1 genome association with H2B during *de novo* infection in HFF cells was also performed. We observed the association of H2B with EdU-labeled KSHV and HSV-1 genomes early during infection (Fig 9A and 9B, lane 2). DNA purified from unlabeled or EdU-labeled KSHV or HSV-1 infected cells showed similar levels and served as input controls (S7A and S7B Fig, lanes 1 and 2). Upon streptavidin capture, DNA recovery was observed only from cells infected with EdU-labeled virus (S7A and S7B Fig, lane 4) but not from cells infected with unlabeled virus (S7A and S7B Fig, lane 3). This demonstrated the specificity of the EdU genome pull down assay.

Our recent studies showed that BRCA1 knockdown reduced the association of IFI16 with KSHV and HSV-1 genomes [1]. To determine the role of H2B in KSHV genome association by IFI16, HMVEC-d cells transfected by siC, siH2B or siBRCA1 were infected with EdU-genome labeled KSHV and tested by PLA with anti-IFI16 (mouse and goat) antibodies and EdU-labeled genome was detected by an EdU reagent kit [1]. PLA results showed a considerable amount of EdU KSHV (red dots) and IFI16-IFI16 (green dots) association in the nucleus (Fig 9C, top right panel, white arrows and Fig 9D) which were reduced significantly in siH2B infected cells (Fig 9C, middle right panel, white arrows and Fig 9D). This suggested that H2B has a significant role in KSHV genome recognition by IFI16. Similarly, in PLA reactions to detect the EdU-labeled HSV-1 genome association with IFI16 during *de novo* HFF cell infection we observed substantial levels of EdU-HSV-1 and IFI16-IFI16 association in the nucleus (Fig 9E, top right panel, white arrows and Fig 9F) which was reduced significantly in siH2B infected cells (Fig 9E, middle right panel, white arrows and Fig 9F). In addition, we observed significantly less IFI16 distribution in the cytoplasm of HMVEC-d and HFF cells infected by KSHV and HSV-1 in siH2B compared to siC infected cells (Fig 9C and 9E, red arrows left panels). siBRCA1 was used as a positive control [1] and we observed substantial reduction in IFI16 association with KSHV and HSV-1 genomes (Fig 9C–9F).

Collectively, these observations revealed the H2B association with the KSHV and HSV-1 genomes and highlighted the essential role of H2B in the regulation of KSHV and HSV-1 genome recognition by IFI16 and correlated to the subsequent IFI16 mediated host innate IFN-β responses.

### H2B plays an important role in association of IFI16 with HSV-1 genome

To define the association of H2B with viral genomes further, HFF cells electroporated with siC, siH2B or siBRCA1 were infected with unlabeled or EdU-labeled HSV-1 (10 pfu/cell) for 2 h and then protein-DNA cross-linking was performed as described in Fig 9A. The purified DNA from unlabeled or EdU-labeled HSV-1 infected cells showed similar levels in siC, siBRCA1 and siH2B electroporated cells (S7C Fig, lanes 8–13). However, DNA was recovered by streptavidin capture materials only from EdU-labeled HSV-1 infected cells (S7C Fig, lanes 5–7) but not from unlabeled virus infected cells (S7C Fig, lanes 2–4). DNA shearing and short chromatin fragments were captured on streptavidin beads and pull down proteins were analyzed by immunoblotting using anti-IFI16 and H2B antibodies. The absence of any protein in unlabeled HSV-1 infected cells validated the specificity of these reactions (Fig 9G, top two panels, lanes 1–3). We observed substantial levels of IFI16 association with HSV-1 genome (siC) which was significantly reduced by siBRCA1 (80%) as well as in siH2B (59%) infected cells (Fig 9G, top panel, lanes 4–6). H2B association with viral genome was significantly reduced (79%) in the siH2B cells and in contrast, we observed a substantial level of H2B association with HSV-1 genome in siBRCA1 with only ~8% less compared to siC cells (Fig 9G, second panel, lanes 4–6).

This suggested that H2B associates with HSV-1 genome independent of its innate response functions mediated through its association with IFI16 and BRCA1. Nevertheless, collectively our results suggested that H2B is critical in KSHV and HSV-1 genome recognition by IFI16 during *de novo* infection.

### H2B-IFI16 association with KSHV genome during *de novo* infection of HMVEC-d cells

Since we observed that H2B associates with IFI16 in the nucleus of uninfected and KSHV infected cells (Figs 1 and 2) and H2B was also pulled down by KSHV and HSV-1 genome during *de novo* infection (Fig 9A and 9B), we next determined whether H2B participates in viral genome recognition by IFI16. HMVEC-d cells transfected with siC and siBRCA1 followed by infection with EdU-labeled KSHV were subjected to PLA reaction using anti-IFI16 (mouse) and anti-H2B (rabbit) antibodies. PLA revealed association of IFI16-H2B (green dots) and EdU-KSHV (red dots) in the nucleus of cells which were significantly reduced in BRCA1 knockdown cells (Fig 9H). The average colocalized PLA spots (yellow color) per cell are presented in the bar graphs (Fig 9I). These results suggested that IFI16 in complex with H2B associates with the KSHV genome and BRCA1 participates in this association.

### IFI16 and H2B acetylation depends upon BRCA1 during KSHV *de novo* infection

**Fig 10:**
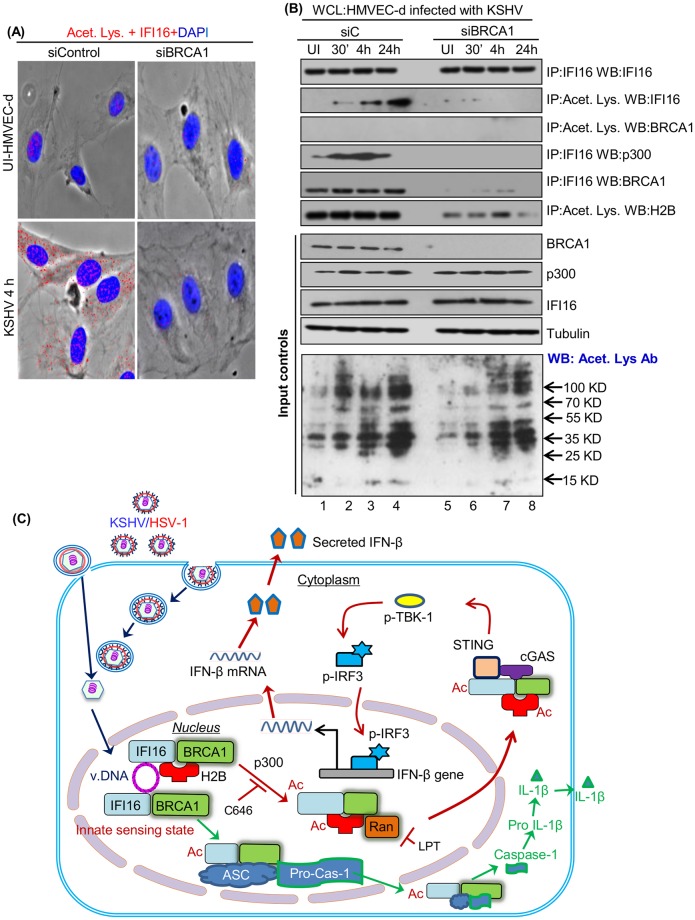
Effect of BRCA1 on IFI16 and H2B acetylation during KSHV *de novo* infection. (A) HMVEC-d cells electroporated with siC or siBRCA1 for 48 h were uninfected or infected by KSHV for 4 h and subjected to PLA using mouse anti-IFI16 and rabbit anti-acetyl lysine antibodies. The localization of IFI16-acetyl lysine (acetylation of IFI16) PLA spots are shown in red. (B) WCL from HMVEC-d cells electroporated with siC and siBRCA1 for 48 h followed by with or without KSHV infection for 30’ (30 min), 4 and 24 h were IP-ed using anti-IFI16 and anti-acetyl lysine antibodies and western blotted for IFI16, BRCA1, p300, and H2B. Bottom input control panels show the levels of BRCA1, IFI16, p300 and total protein acetylation. (C) Schematic model illustrating the essential role of histone H2B in IFI16-mediated viral DNA genome sensing and innate IFN-β production during KSHV or HSV-1 *de novo* infection. Soon after KSHV or HSV-1 DNA entry into the nucleus, IFI16 in complex with BRCA1-H2B or with BRCA1 recognizes the viral genome, leading into BRCA1 mediated p300 recruitment, interaction with IFI16, acetylation (Ac) of IFI16 and H2B by p300, and cytoplasmic transport of acetylated IFI16-H2B-BRCA1 via Ran GTP. C646 or LPT treatments abolish acetylation or cytoplasmic translocation of H2B and IFI16, respectively. The inflammasome (IFI16-BRCA1-ASC-procaspase-1) independent IFI16-H2B-BRCA1 complex in the cytoplasm associates with cGAS and STING to form a macromolecular complex leading into phosphorylation of TBK1 and IRF3, nuclear translocation of p-IRF3 and subsequent IFN-β production during KSHV or HSV-1 *de novo* infection. Viral genome recognition by IFI16-BRCA1 results in acetylation of IFI16 which interacts with ASC and procaspase-1 in the nucleus. This BRCA1-IFI16-ASC-procaspase-1 complex is transported to the cytoplasm via Ran-GTP resulting in caspase-1 formation, pro-IL-1β cleavage and IL-1β formation [1, 9]. These studies demonstrate that the innate nuclear foreign viral genome sensing responses are mediated by two IFI16 complexes, with recognition by the IFI16-H2B-BRCA1 complex resulting in IFN-β responses while recognition by the IFI16-BRCA1 complex results in inflammasome-IL-1β responses.

Our studies show that KSHV induces IFI16 and p300 interaction and p300 is required for KSHV induced acetylation of IFI16 [9], and that BRCA1 knockdown results in inhibition of IFI16 translocation to the cytoplasm, and subsequent IL-1β and IFN-β induction [1]. Since KSHV or HSV-1 genome recognition by IFI16 alone or in complex with H2B (Fig 9) was significantly reduced in the absence of BRCA1 and since BRCA1 is known to interact with p300 [24], we hypothesized that besides involvement in IFI16’s ability to recognize viral episomal genomes, BRCA1 may also be involved in the post-genome recognition event of acetylation of IFI16 and H2B. To determine the role of BRCA1 in the acetylation of IFI16, HMVEC-d cells electroporated with siC and siBRCA1 were left uninfected or infected with KSHV for 4 h and tested by PLA reactions using anti-IFI16 and anti-acetyl lysine antibodies. PLA analysis revealed that BRCA1 knockdown abolished the acetylation of IFI16 during KSHV *de novo* infection (Fig 10A).

To further verify the effect of BRCA1 on H2B and IFI16 acetylation, HMVEC-d cells electroporated with siC and siBRCA1 were left uninfected or infected by KSHV for different time points and WCL was IP-ed using anti-IFI16 and, anti-acetyl lysine antibodies and western blotted for IFI16, BRCA1, p300 and H2B. The IP results demonstrated that IFI16 and p300 interaction as well as IFI16 and H2B acetylation were inhibited in BRCA1 knockdown cells during KSHV *de novo* infection (Fig 10B). Bottom panels showed input controls for BRCA1, p300, IFI16 and total protein acetylation levels (Fig 10B, bottom panels). Collectively, these results demonstrated that BRCA1 is not only essential for genome recognition by IFI16 alone or in complex with H2B but also plays important roles in the recruitment of p300 for the acetylation of IFI16 and H2B, which leads into IFI16 interaction with ASC, inflammasome formation, transport into the cytoplasm, and IL-1β induction as well as inflammasome independent IFI16-H2B translocation into the cytoplasm, interaction with cGAS-STING and IFN-β induction.

## Discussion

The innate immune response is a very effective front line host defense against microbial pathogens including viruses. Eukaryotic nuclear proteins, besides being involved in localized functions, also mediate functions in the cytoplasm. For example, high mobility box protein 1 (HMGB1) involved in transcriptional regulation and DNA organization also acts as an “alarmin”, exits the nucleus during necrosis and activates innate immune signaling by binding to viral RNAs and DNAs [25]. Extrachromosomal functions of histones have also been observed. An apoptotic stimulus, such as DNA damage induces the translocation of nuclear histone H1.2 to mitochondria by an unknown mechanism to promote the mitochondrial apoptotic pathway [26]. Extrachromosmal H2B was shown to be one of the potential mediators of the IFN-β response to cytoplasmic host DNA [17]. Our studies not only identify H2B as an innate immune sensor of nuclear herpesviral genomes but also define the potential mechanisms by which H2B mediates its extrachromosomal IFN-β response function during herpes viral infection.

Our comprehensive studies (Fig 10C) demonstrate for the first time that: a) histone H2B is in a complex with innate immune DNA sensor IFI16 and BRCA1 proteins in the nucleus. This is independent of its interactions with histone H2A. b) H2B is a component of IFI16-BRCA1, and sensing of the nuclear herpesvirus episomal genome results in BRCA1 dependent recruitment of p300 to IFI16 and subsequent acetylation of H2B and IFI16. c) Acetylated IFI16-H2B in association with BRCA1 is exported to the cytoplasm by Ran-GTP where they interact with cGAS and STING, resulting in pIRF3 induction and IFN-β production. d) Independent of H2B-IFI16-BRCA1, genome recognition by IFI16-BRCA1 leads to the formation of a BRCA1-acetylated IFI16-ASC-procaspase-1 inflammasome complex, which also translocates to the cytoplasm resulting in caspase-1 formation and subsequent cleavage of pro-IL-1β, and e) Independent of its interactions with IFI16-BRCA1, H2B associates with viral genome.

We have shown that BRCA1 is associated with IFI16 in uninfected cells which increased in KSHV, HSV-1 and EBV infected cells. Also, BRCA1 is part of the IFI16-ASC-procaspase-1 inflammasome complex [1]. BRCA1 is essential for KSHV and HSV-1 genome recognition by IFI16 since in the absence of BRCA1, IFI16’s association with the viral genome is significantly reduced (Fig 9) [1], resulting in decreased IFI16 cytoplasmic translocation, and inflammasome as well as IFN-β responses [1]. Significant reduction in IFI16’s association with viral genome (Fig 9) in H2B knockdown cells and the near absence of an IFN-β response suggests that H2B, in association with IFI16-BRCA1, is involved in viral genome sensing. However, results such as ~40% association of IFI16 with viral genome (Fig 9G) and induction of IL-1β secretion in the infected cells in the absence of H2B (Fig 7D) demonstrate that the function of viral genome recognition resulting in BRCA1-IFI16-ASC-procaspase-1 inflammasome complex formation is not affected.

The IFI16-H2B complex is mainly involved in inflammasome independent STING-mediated IFN-β production as shown by the association of cytoplasmic IFI16-H2B-BRCA1 complex with cGAS and STING to form a signal hub and IFN-β production (Fig 10C), absence of IFI16-STING association in H2B knockdown cells during KSHV *de novo* infection (Fig 6G), absence of H2B interaction with ASC (Fig 2B and 2D) and absence of STING interaction with ASC (S6C–S6E Fig). These observations are consistent with earlier studies demonstrating that ASC is not essential for IFN-β production [6, 8]. Therefore, we surmise that nuclear viral genome sensing is mediated by at least two IFI16 complexes in which 1) recognition by the IFI16-BRCA1-H2B complex results in the IFN-β response, and 2) recognition by the IFI16-BRCA1 complex results in interactions with ASC and inflammasome formation.

The BRCA1-IFI16 complex that we observed is not related to the host DDR responses induced by the addition of bleomycin in the uninfected HMVEC-d cells [1]. Though IFI16 association with viral genome was significantly reduced in the absence of BRCA1, H2B association with viral genome was not affected (Fig 9G). This suggests that independent of BRCA1 and IFI16, H2B associates with viral genome probably to mediate its nucleosome associated functions, which are distinct from the IFI16-BRCA1-H2B mediated innate interferon response. This is supported by our earlier observations that there was no significant IFN-β production in the BCBL-1 cells [1,3] which could be due to a virus strategy to avoid antiviral effects as several viral latent proteins such as LANA-1 and vIRF-1 have been shown to block the pIRF nuclear functions of IFN response gene activation [27–29].

Nuclear histone H2A, H2B, H3 and H4 proteins form octamers, bind and package DNA into ordered nucleosome units consisting of two H2A-H2B dimers and an H3-H4 tetramer [30–32]. H2B undergoes several modifications such as acetylation, phosphorylation and ubiquitination that are essential for its various roles, and p300 is essential for the acetylation of H2B-H2A [16, 18, 19, 33–37]. Our studies demonstrate that basal levels of H2B acetylation in the nucleus increased in KSHV infected cells and acetylation of IFI16 associated H2B is crucial for H2B’s cytoplasmic translocation along with IFI16 and BRCA1 and the consequent innate IFN-β response. Nuclear herpes viral genome recognition by IFI16 is dependent upon its association with BRCA1 but independent of its acetylation [9]. Increased IFI16-p300 interaction was observed only after nuclear herpes viral genome entry resulting in increased p300 activity only in the nucleus. This increased acetylation was not due to decreased activity of HDACs [9]. We speculated a role in IFI16-p300 interaction as BRCA1 is known to interact with p300 [24]. Absence of IFI16 interaction with p300 together with the absence of increased IFI16 and H2B acetylation in BRCA1 knockdown cells (Fig 10B) clearly demonstrate that besides its role in IFI16’s ability to recognize the episomal viral genomes [1], BRCA1 is essential for the recruitment of p300 to the IFI16-H2B-BRCA1 complex leading to H2B and IFI16 acetylation.

p300-mediated acetylation has been shown to modify the nucleosome structure to facilitate the disassociation and transfer of H2B-H2A from the nucleosome to histone chaperon NAP-1 [37]. Even though H2B interactions with H2A were observed in the nucleus of uninfected and infected cells, only H2B in association with IFI16 and BRCA1 was detected in the cytoplasm of virus infected cells (Figs 1 and 2). It is possible that acetylation of IFI16 and H2B probably leads to changes in their affinity and structure to facilitate their dissociations from the viral DNA leading to their association with RAN-GTP, transport to the cytoplasm leading into interaction with cGAS and STING then IFN-β production (Fig 10C). The detection of IFI16 association with the viral genomes in KSHV and EBV in latently infected cells suggest that recognition, acetylation and relocalization of H2B-IFI16 is a dynamic continuous event with IFI16 always occupying the viral genomes [3, 9].

KSHV doesn’t infect laboratory animals including primates. We have examined human tissue sections from normal skin, KSHV+ Kaposi’s sarcoma (KS) lesions, control lung and KSHV+ solid lung primary effusion B-cell lymphoma (PEL) lesions. IFI16 and ASC colocalization was not observed in the control skin and normal lung sections. In contrast, perinuclear cytoplasmic colocalization of ASC and IFI16 was observed in KSHV+ KS and PEL lesions [3]. These studies demonstrated the potential *in vivo* involvement of the IFI16-inflammasome in KSHV biology.

cGAS, identified as a cytoplasmic DNA sensor [11, 22, 38–41], was also detected in the nucleus and cytoplasm of HFF cells and immortalized oral keratinocyte cells [10], and in the nucleus and cytoplasm of BCBL-1 cells. However, IFI16 is the primary sensor of HSV-1 DNA in the nucleus and cGAS is not involved in genome recognition [10]. cGAS is believed to stabilize IFI16 in HSV-1 infected cells [10]. However, it is not clear whether this interaction or stabilization occurs in the nucleus or in the cytoplasm. Nevertheless, cGAS and IFI16 knockdown impaired the IFN-β responses in HSV-1 infected HFF cells [10]. We also observed similar findings (Fig 7) and in addition, demonstrate the interactions of cGAS with H2B, IFI16, BRCA1, and STING in the cytoplasm of cells during *de novo* infection as well as in latent infection (Figs 5O, 5Q and 6). A recent study also demonstrated the Interaction of cGAS with STING in the cytoplasm during *Chlamydia tracomatis* infection resulting in IFN-β production [42].

Our earlier studies have shown that the IFI16-inflammsome is not induced by the infection of HMVEC-d cells with lentivirus expressing KSHV proteins [2] which suggested that IFI16 perhaps doesn’t recognize the integrated lentvirus genome as foreign. A recent study with HCMV (Ad169) infection for 6 h in IFI16 knockout (KO) human fibroblast cells suggests that IFI16 is not necessary for the IFN-β response [43]. However, as responses against HSV-1 or KSHV *de novo* infections are not examined in these studies under similar IFI16 KO conditions, it is premature to conclude the role of IFI16 in the IFN-β response during the complex biology of various herpesvirus infections. Moreover, our ongoing studies with HSV-2 and HSV-1 with human osteosarcoma cells in which IFI16 is knocked out by CRISPR [6] demonstrate that in the absence of IFI16, the IFN-β response is significantly abrogated.

IFI16, H2B, BRCA1 and cGAS knockdown clearly demonstrate that a macromolecular complex of these molecules is necessary for STING activation and innate IFN-β response during KSHV and HSV-1 *de novo* infection (Fig 7). Moreover, H2B knockdown significantly reduced the IFI16-STING and IFI16-cGAS association and cGAMP production (Figs 6, 7 and 8). Furthermore, THP-1-Dual KO-STING cells results (Fig 8B2) confirmed that IFN-β induction is mainly mediated through the cGAS-STING pathway which demonstrates that H2B is essential for the IFI16-H2B-cGAS-STING-mediated IFN-β response. Whether cGAS stabilizes IFI16 in complex with STING and whether cGAS forms single or separate complex with H2B and IFI16 needs to be studied thoroughly which are beyond the scope of our present study. Similarly, the role of nuclear cGAS in the IFI16-STING-mediated innate IFN-β response requires additional studies.

## Materials and Methods

### Reagents

Leptomycin B (LPT), EdU (5-ethynyl-2’-deoxyuridine), and C646 (Sigma-Aldrich), SlowFade Gold Antifade reagent with DAPI (Life Technologies), Verikine human IFN-β ELISA kit (PBL Assay Science) and IL-1β ELISA kit were from RayBiotech, Inc.

### Cells

Human dermal microvascular endothelial (HMVEC-d) cells and human foreskin fibroblast (HFF) cells (Clonetics), BJAB and BCBL-1 cells (ATCC CRL 8799 and 2336) were grown as described earlier [2, 4, 5].

### Virus preparation and infection

KSHV was purified from the supernatant of induced BCBL-1 cells using phorbol ester and virus DNA copy number was analyzed by real-time DNA-PCR [2]. In most of the experiments KSHV infection was done with 30 DNA copies/cell for 2 h in serum free medium, washed, and then replaced with complete medium for different time points of infection [2].

HSV-1 (KOS) production and viral titer using a plaque assay on Vero cells were performed as described earlier [4]. In most of the experiments, HSV-1 infection was done with 1 pfu/cell (~25 DNA copies/cell) in serum free medium for 2 h, washed and replaced with complete medium and incubated for other time points.

### Antibodies

Antibodies are listed in Table 1. The secondary antibodies conjugated to HRP against anti-rabbit, anti-goat and anti-mouse IgG and Alexa Fluor-488, and -594 (Molecular Probes) and VeriBlot for IP secondary antibody (HRP) were purchased from Abcam.

**Table 1:** List of antibodies used in this study.

Antibody	Species	Source
Histone H2B (sc10808)	Rabbit polyclonal	Santa Cruz Biotechnology, Inc., Santa Cruz, CA
Histone H2B (sc 8650)	Goat polyclonal	Santa Cruz Biotechnology, Inc., Santa Cruz, CA
Histone H2B (12364S)	Rabbit monoclonal	Cell Signaling Technology, Beverly, MA
Histone H3 (4499P)	Rabbit monoclonal	Cell Signaling Technology, Beverly, MA
Histone H2A (sc 8648)	Goat polyclonal	Santa Cruz Biotechnology, Inc., Santa Cruz, CA
Histone H2A (2578S)	Rabbit monoclonal	Cell Signaling Technology, Beverly, MA
BRCA1 (GTX70111)	Mouse monoclonal	GeneTex, Irvine, CA
BRCA1 (A301-377A)	Rabbit polyclonal	Millipore, Billerica, MA
ASC (D086-3)	Mouse monoclonal	MBL International, Woburn, MA
ASC/TMS1 (ER-03-0001)	Goat polyclonal	Ray Biotech, Norcross, GA
IFI16 (SC8023)	Mouse monoclonal	Santa Cruz Biotechnology, Inc., Santa Cruz, CA
IFI16 (SC6050)	Goat polyclonal	Santa Cruz Biotechnology, Inc., Santa Cruz, CA
IFI16 (HPA002134)	Rabbit polyclonal	SIGMA, St Louis, MO
p-TBK-1 (D52C2)	Rabbit monoclonal	Cell Signaling Technology, Beverly, MA
TBK-1/NAK (3013)	Rabbit	Cell Signaling Technology, Beverly, MA
Ran (ab4781)	Rabbit polyclonal	Abcam Inc., Cambridge, MA
cGAS (15102S)	Rabbit monoclonal	Cell Signaling Technology, Beverly, MA
cGAS (sc245858)	Goat polyclonal	Santa Cruz Biotechnology, Inc., Santa Cruz, CA
STING (13647S)	Rabbit monoclonal	Cell Signaling Technology, Beverly, MA
p-IRF-3 (29047S)	Rabbit monoclonal	Cell Signaling Technology, Beverly, MA
IRF-3 (ab50772)	Mouse monoclonal	Abcam Inc., Cambridge, MA
P300 (sc585)	Rabbit polyclonal	Santa Cruz Biotechnology, Inc., Santa Cruz, CA
β-Actin (A5441)	Mouse monoclonal	SIGMA, St Louis, MO
β-Tubulin (T0198)	Mouse monoclonal	SIGMA, St Louis, MO
TBP (ab51841)	Mouse monoclonal	Abcam Inc., Cambridge, MA
Acetylated-Lysine (9441S)	Rabbit polyclonal	Cell Signaling Technology, Beverly, MA
Alexa 594 (A11037; A11005)	Rabbit or Mouse	Molecular Probes, Invitrogen, Carlsbad, CA
Alexa 488 (A11034; A11029)	Rabbit or Mouse	Molecular Probes, Invitrogen, Carlsbad, CA
HRP tagged secondary antibody	Rabbit (074–15) or Mouse (074–1806) or goat (14-13-06)	KPL Inc., Gaithersburg, MD

### EdU labeled KSHV and HSV-1 genome

The BCBL-1 cells were induced using phorbol ester and KSHV DNA was labeled by adding EdU (5-ethynyl-2’-deoxyuridine) (10 μM) in the culture medium during lytic replication on the first and third day of induction [1]. The labeled viruses from the cell culture supernatant (day 5) were purified and their genome copy number was analyzed by real-time DNA-PCR [2]. HSV-1 (KOS) genome was labeled by adding EdU to the Vero cell medium at 8, 24 and 48 h post-infection [1]. On day 4, the culture supernatant was collected and labeled virus was purified and titrated [4].

### Preparation of nuclear and cytoplasmic fractions

Cells were harvested and used for preparation of nuclear and cytoplasmic extracts using a nuclear complex Co-IP kit (Active Motif Corp.). Nuclear and cytoplasmic proteins were estimated using BCA protein assay reagent (Pierce), and purity of the fractions was determined by western blotting using anti-TBP and anti-β-tubulin antibodies, respectively.

### Western blot analysis and immunoprecipitation (IP)

Cells were lysed in RIPA (radioimmunoprecipitation assay) lysis buffer (15 mM NaCl, 1 mM MnCl2, 1 mM MgCl2, 2 mM phenylmethylsulfonyl fluoride plus protease inhibitor cocktail), sonicated, and centrifuged at 10,000 rpm at 4°C for 10 min for western blot analysis. An equal amount of proteins were separated by SDS-PAGE, transferred to nitrocellulose and incubated with primary antibodies followed by HRP-conjugated secondary antibodies. Immunoreactive protein bands were detected by chemiluminescence (Pierce) as per manufacturer’s instructions. For IP, the harvested cells were lysed using IP lysis buffer (25 mM Tris-HCl, pH7.5, 150 mM NaCl, 1% NP40, 2 mM EDTA, 10% Glycerol, and protease inhibitor mixture) and 150 to 200 μg of precleared whole cell lysates or extracted nuclear/cytoplasmic fractions were incubated overnight with primary antibodies at 4°C. The immune complexes were captured using protein A- or G-sepharose (GE Healthcare, PA), washed thrice and examined by immunoblotting. Blots were scanned by an AlphaImager system (Alpha Innotech Corp.) and quantitated by ImageJ software.

### RNA interference by electroporation

Primary HMVEC-d and HFF cells were electroporated with different siRNAs using a Neon Transfection System (Invitrogen) as per manufacturer’s instructions [1]. Briefly, subconfluent monolayer cells were harvested and washed with 1X PBS (phosphate-buffered saline) and resuspended (1x10^7^ cells/ml) in resuspension buffer R (Invitrogen). Ten microliters of cell suspension plus 100 pmol of siRNA were mixed and then used for microporation at room temperature using a single pulse of 1350 V for 30 ms for HMVEC-d and 1700 V for 20 ms for HFF cells. Soon after microporation, cells were dispersed into complete medium and incubated at 37°C in a humidified 5% CO2 incubator. After 48 h electroporation, cells were used either for nuclear or cytoplasmic fractions or lysed in IP or RIPA buffer and knockdown efficiency was determined by immunoblots. siRNA oligonucleotides for BRCA1 and IFI16 (siGenome SMART pool), STING (smart pool: siGenome TMEM173), ASC and C6orf150 (cGAS) (Santa Cruz Biotechnology, Inc) and a non-targeting siRNA pool were purchased from Thermo Scientific. For H2B siRNA, dsRNA was synthesized by Invitrogen (stealth RNAi): histone H2B sense, 5′-UCC AAG GCC AUG GGC AUC AUG AAC U-3′; histone H2B antisense, 5′-AGU UCA UGA UGC CCA UGG CCU UGG A-3′. The non-coding stealth siRNA was purchased from Invitrogen.

### Immunofluorescence microscopy

Primary HMVEC-d and HFF cells seeded on glass chamber slides (Nalgene Nunc International) were uninfected, KSHV infected (30 DNA copies/cell), or HSV-1 infected (1pfu/cell), fixed for 15 min with 4% paraformaldehyde, and permeabilized using 0.2% Triton X-100 for 5 min. Cells were then washed and blocked using Image-iT signal enhancer (Life Technologies) for ~20 min followed by incubation with primary antibodies and then incubated with secondary antibodies conjugated with fluorescent dye. To detect EdU labeled viral genome, cells were fixed, permeabilized and blocked with Image-iT signal enhancer for 20 min. A CLICK reaction was performed for 30 min at RT using Click-iT EdU reaction additive (Life Technologies), copper sulphate, EdU reaction buffer and Alexa Fluor 594 azide. Cells were observed by Nikon Eclipse 80i microscope, and analyzed with Metamorph digital imaging software. All images were acquired at 40X magnification.

### IFN-β and IL-1β ELISA

Cell culture supernatants from uninfected or virus infected cells (~3X10^5^) were collected and levels of IFN-β and IL-1β secretion were measured [2, 6]. The absorbance was read at 450 nm using a Synergy2 Biotek Plate Reader (Biotek).

### *In situ* proximity ligation assay (PLA) microscopy

Protein—protein interactions were studied using a DUOLink PLA kit (Sigma) as described earlier [1]. Briefly, uninfected and KSHV (30 DNA copies/cell) infected HMVEC-d cells or HSV-1 (1 pfu/cell) infected HFF cells were seeded in chamber microscope slides, fixed with 4% PFA for 15 min, permeabilized using 0.2% Triton X-100 for 5 min and then blocked with blocking buffer for 30 min at 37°C. For BJAB and BCBL-1 cells, equal numbers of cells were washed with PBS by centrifugation at 200xg at 4°C and spotted on glass slides, then fixed/permeabilized with pre-chilled acetone and blocked with blocking buffer. Cells were incubated with primary antibodies, washed and further incubated with species specific PLA probes (PLUS and MINUS probes) under hybridization conditions in the presence of two additional oligonucleotides to enable hybridization of PLA probes if they were in proximity of <40 nm. A ligation mixture was added to form a closed circle while amplification mixtures result in the formation of a concatemeric product extending from the oligonucleotide arm of the PLA probe. Finally, a detection mixture consisting of fluorescently labeled oligonucleotides was added, and the labeled oligonucleotides were hybridized to the concatemeric products. The signal was detected as a distinct fluorescent dot in the FITC green or Texas red channel and analyzed by fluorescence microscopy. Negative controls consisted of samples treated as described above but with only primary, secondary or control IgG antibodies. The average number of PLA dots per cell was quantified using DUOLink software.

For double PLA, two independent PLA reactions were performed sequentially [1]. Briefly, the PLA reaction for IFI16, H2B and STING was performed first using mouse anti-IFI16 and goat anti-H2B antibodies and detected by DUOLink green detection agent. Cells were then washed, blocked and subjected to a second PLA reaction with goat anti-H2B and rabbit anti-STING antibodies and detected with DUOLink red detection agent.

### cGAMP production

HMVEC-d cells electroporated for 48 h with siC, siH2B and siIFI16 were left uninfected or infected with KSHV (30 DNA copies/cell) for 4 h. Cells were lysed, treated with benzonase for 30 min at 37°C, and heat inactivated at 95°C for 5 min. 10 μl of heat inactivated lysates or varying amounts of purified cGAMP were added to the 1X10^5^ THP-1-Lucia ISG (InVivoGen) and THP-1-Dual KO-STING cells (InVivoGen) pretreated with digitonin for 30 minutes. Cells were then incubated overnight, and 10 μl culture supernatant mixed with 50 μl of QUANTI-Luc luminescence assay solution (InVivogen, San Diego, CA) and cGAMP level assayed by the luminescence read on an ELISA plate reader.

### EdU labeled KSHV or HSV-1 viral DNA mediated chromatin pull down assay

The EdU-labeled viral genome (chromatin) pull down method has been described earlier [1]. Briefly, HMVEC-d and HFF cells (~8x10^6^ cells/ml) with or without control H2B or BRCA1 siRNA for 48 h were infected with unlabeled or EdU labeled KSHV (200 DNA copies/cell) and HSV-1 (10 pfu/cell) for 2 h and then cross-linked with 1% formaldehyde for 10 min at 4°C. Unreacted formaldehyde was quenched using 0.125 M glycine for 10 min at 4°C and cells were harvested, permeabilized (0.1% Triton X-100) for 10 min and washed with PBS. Biotin was linked to EdU genome by a Click reaction using sequential addition of (+)-sodium-L-ascorbate (10 mM), biotin-TEZ azide (0.1 mM) and copper (II) sulfate (2 mM) for 30 min in the dark followed by adding 1% BSA and 0.5% Tween 20 for 10 min. The soluble proteins were isolated in 500 μl CL lysis buffer (50 mM HEPES, pH 7.8, 0.25% Triton X-100, 0.5% NP-40, 150 mM NaCl, 10% glycerol plus protease inhibitors) for 10 min at 4°C and centrifuged at 300xg. The pellet containing chromatin-protein complexes was washed with wash buffer (10 mM Tris-HCL, pH 8.0, 0.5 mM DTT, 200 mM NaCl) at 4°C for 10 min and then resuspended in 500 μl RIPA buffer (10 mM Tris-HCl, pH 8.0, 0.1% Na-Deoxycholate, 0.1% SDS, 1% Triton X-100 and 140 mM NaCl plus protease inhibitor cocktail) and chromatin was sheared by sonication. The sonicated extract was clarified by centrifugation (15,000xg) for 10 min at 4°C and 1 mg of the extract was used for pull down using 50 μl of streptavidin magnetic beads. Beads with bound complexes were subjected to reverse protein-DNA cross-linking and proteins were eluted in 1X Laemmli sample buffer (95°C for 10 min) for immunoblotting. To purify DNA, the complexes were eluted from beads in elution buffer (0.1 M NaHCO3 and 1% SDS) and cross-linking was reversed by treating with 0.1 mg/ml RNase A and 0.3M NaCl for 30 minutes at 37°C and then incubated at 65°C for 2 h with 0.1 mg/ml Proteinase K. Eventually, DNA was column purified using a Qiagen DNA extraction kit as per manufacturer’s instructions.

### Statistical analysis

Data are expressed with means ± SD of at least three independent experiments using a Student’s T-test. p<0.05 was considered statistically significant.

## Supporting Information

S1 Table Mass spectrometric analysis of uninfected nuclear proteins interacting with IFI16. Nuclear fractions from uninfected HMVEC-d cells were isolated using a Nuclear Complex Co-IP Kit (Active Motif, CA). 100 μg of nuclear fraction was immunoprecipitated overnight at 4°C with anti-IFI16 or IgG control antibodies. Immunoprecipitates were resolved using a 4–20% gradient SDS-PAGE gel (Bio-Rad) and were stained with coomassie brilliant blue (CBB) dye. The bands of interest were analyzed by mass spectrometry (MS) using an LC-ESI (electrospray ionization)-MS based approach at the Midwest Proteome Center, Rosalind Franklin University of Medicine and Sciences. MS analysis revealed several proteins and the six proteins with the highest percentage of PEAKS scores and coverage are shown.
(DOCX)

S1 Fig Proximity ligation assay (PLA) analysis of the association of H2B with H2A and IFI16 with ASC. (A and B) Protein-protein close proximity interactions were detected by a DUOLink PLA kit (Sigma). Uninfected BJAB cells were washed with PBS by centrifugation at 200xg at 4°C and spotted on 10-well glass slides, fixed, permeabilized with pre-chilled acetone, and blocked with DUOLink blocking buffer for 30 min at 37°C. Uninfected HMVEC-d and HFF cells cultured in 8 well chamber microscope slides were fixed, permeabilized and blocked with DUOLink blocking buffer for 30 min at 37°C. Blocked BJAB, HMVEC-d and HFF cells were incubated with primary antibodies, anti-H2B (rabbit), anti-H2A (mouse), anti-IFI16 (rabbit) or anti-ASC (mouse) antibodies for 1 h at 37°C, washed, incubated for 1 h at 37°C with species specific PLA probes (PLUS and MINUS probes), anti-mouse probe (+) and anti-rabbit probe (-), under hybridization conditions in the presence of two additional oligonucleotides to enable hybridization of PLA probes that were in close proximity (<40 nm). A ligation mixture with ligase was added to link the two hybridized oligonucleotides to form a closed circle. Multiple cycles of rolling-circle amplification using the ligated circle as a template were performed by adding an amplification solution to form a concatemeric product extending from the oligonucleotide arm of the PLA probe. Eventually, a detection solution containing fluorescently labeled oligonucleotides was added to hybridize with the concatemeric products. The signal was detected as a distinct fluorescent dot in the Texas red or FITC green channel depending on the probes and analyzed by fluorescence microscopy. The association of H2B with H2A and IFI16 with ASC was observed by green colored dots in the nucleus of the above cells as indicated by red arrows. Nuclei were stained by DAPI and boxed areas were enlarged in the rightmost panels. (C and D) Bar diagrams represent the quantitation of the average number of PLA dots per cell in the cytoplasm and nucleus of uninfected BJAB, HMVEC-d and HFF cells. (E and F) PLA reaction analysis of the association of IFI16 with H2A and H2B with ASC. Uninfected BJAB, HMVEC-d and HFF cells were fixed, permeabilized and blocked in blocking buffer and incubated with primary anti-IFI16 (rabbit), anti-H2A (mouse), anti-H2B (rabbit) or anti-ASC (mouse) antibodies and the PLA reaction was performed as described in figure S1 (panel A and B). Nuclei were stained with DAPI and boxed areas were enlarged in the rightmost panels. PLA analysis revealed no significant localization of IFI16 with H2A and between H2B and ASC in the uninfected cells.
(TIF)

S2 Fig Immunofluorescence (IFA) and PLA analysis during KSHV and Vaccinia virus infection. (A) Specificity controls for PLA reactions. As specificity controls for all PLA reactions, negative controls such as use of a single species primary antibody, secondary antibody alone or control IgG antibody were used to perform the complete PLA process as described in S1A Fig. Magnification: 40X. (B) Localization of IFI16 with H2B by IFA. BJAB and HMVEC-d cells were fixed, permeabilized, blocked in Image-iT signal enhancer, incubated with primary anti-IFI16 and anti-H2B antibodies for 1 h. After washing, these were incubated with secondary antibodies, anti-mouse Alexa Fluor 594 for IFI16 and anti-rabbit Alexa Fluor 488 for H2B, for 1 h. DAPI was used for nuclear staining. Boxed areas were enlarged in the rightmost panels. Red arrows indicate the colocalization of IFI16 with H2B in the nucleus. (C and D) Quantitation of PLA spots of IFI16-H2B during KSHV *de novo* infection. Uninfected HMVEC-d cells were infected for 4 h (C) and 2, 12 and 24 h (D) with KSHV (30 DNA copies/cell) and subjected to PLA reaction using anti-IFI16 (mouse) and H2B (rabbit) antibodies as described in S1A Fig. PLA analysis revealed the association of IFI16 with H2B during KSHV *de novo* infection. The average number of spots per cell in the nucleus and cytoplasm was quantitated and presented in the bar diagram. Magnification: 40X. (E) Localization of IFI16 with H2B during KSHV (KS) *de novo* infection by IFA. HMVEC-d cells were infected by KSHV (30 DNA copies/cell) for 2 h, washed and then incubated in complete medium for various time points (2, 4, 12, 24 h). Uninfected and KSHV infected cells were fixed, permeabilized, blocked, incubated with anti-IFI16 and anti-H2B primary antibodies for 1 h at RT, followed by incubation with secondary antibodies (IFI16:anti-mouse Alexa Fluor 594; H2B:anti-rabbit Alexa Fluor 488) for 1 h. DAPI was used as nuclear stain and the boxed areas from the merged panels were enlarged in the rightmost panels. White and red arrows represent localization of IFI16 with H2B in the nucleus and cytoplasm, respectively. IFA results showed increased localization of IFI16 with H2B at 2, 4 and 12 h p.i. which was reduced at 24 h KSHV p.i. (F) PLA analysis of IFI16 association with H2A during KSHV *de novo* infection. HMVEC-d cells were infected with KSHV (30 DNA copies/cell) for 2 h, washed and incubated in complete medium for 2 h (total 4 h p.i.). The cells were permeabilized, blocked and subjected to PLA reaction using primary anti-IFI16 and anti-H2A antibodies as per S1 Fig. Boxed areas were enlarged. PLA analysis did not show any localization of IFI16-H2A (red dots). (G and H) PLA analysis for H2B-IFI16 or H2B-H2B during vaccinia virus infection in HMVEC-d cells. HMVEC-d cells infected with vaccinia virus (5 pfu/cell) for 4 h were permeabilized and subjected to PLA reaction using anti-H2B (rabbit and goat) and anti-IFI16 (mouse) antibodies as described in S1 Fig. PLA analysis revealed the associations of H2B-IFI16 or H2B-H2B only in the nucleus of uninfected and infected cells, and the few dots seen in the cytoplasm probably represent the basal level of the above associations in uninfected and infected cells.
(TIF)

S3 Fig PLA and IFA analysis for IFI16 and ASC association during KSHV *de novo* infection. (A) HMVEC-d cells were infected by KSHV for 2, 12 and 24 h and PLA reaction was completed using anti-IFI16 and anti-ASC primary antibodies. PLA results showed a few red dots of IFI16-ASC in the nucleus of uninfected cells which profoundly increased in the nucleus as well as in the cytoplasm of cells at 2, 12 and 24 h post-KSHV infection. (B) Average numbers of FI16-ASC PLA red dots in the nucleus and cytoplasm per cell were quantitated and presented in the bar graph. Nucleus vs. cytoplasm dots statistics: * p<0.05, ** p<0.01, NS: not significant. (C) Localization of IFI16 with ASC during KSHV (KS) *de novo* infection by IFA. HMVEC-d cells were infected by KSHV (30 DNA copies/cell), permeabilized as described in S2E Fig, immunostained with anti-IFI16 and anti-ASC primary antibodies for 1 h at RT, and followed by incubation with secondary antibodies (IFI16-anti-mouse Alexa Fluor 594; ASC-anti-goat Alexa Fluor 488). DAPI was used as nuclear stain and the boxed areas from merged panels were enlarged in the rightmost panels. White and red arrows represent localization of IFI16 with ASC in the nucleus and cytoplasm, respectively. Magnification: 40X.
(TIF)

S4 Fig Immunofluorescence analysis of the localization of IFI16-H2B, H2B-BRCA1 and IFI16-BRCA1 in cells latently infected with EBV. (A-C) EBV (-) BJAB and EBV (+) LCL (latency III) and EBV (+) Akata (latency I) cells were washed with PBS, spotted on glass slides, fixed/permeabilized with pre-chilled acetone, blocked using Image-iT signal enhancer for ~20 min, incubated with primary anti-IFI16 (mouse), anti-H2B (rabbit or goat) or anti-BRCA1 (rabbit) antibodies and then secondary antibodies as described in S2E Fig. DAPI was used as nuclear stain and the boxed areas from merged panels were enlarged in the rightmost panels. White and red arrows represent localization in the nucleus and cytoplasm, respectively. Magnification: 40X.
(TIF)

S5 Fig PLA analysis for the detection of IFI16 acetylation during live KSHV *de novo* infection and H2B+STING and IFI16+STING interactions during live and UV- KSHV *de novo* infection. (A and B) PLA analysis for the detection of IFI16 acetylation during KSHV *de novo* infection. Untreated (UT) HMVEC-d cells or cells pre-incubated with p300 inhibitor C646 (1 μM) for 2 h were infected with KSHV (30 DNA copies/cell) for 2 h, washed, and incubated with complete medium for 2 more hours with or without C646. These cells were processed for PLA reactions as in S1 Fig using anti-acetyl lysine (rabbit) and anti-IFI16 (mouse or rabbit) antibodies. Compared to uninfected cells, PLA analysis revealed substantial localization of IFI16 with acetyl lysine in the nucleus and cytoplasm (red dots) of KSHV infected (UT-untreated) cells which was significantly reduced in the presence of C646 (A). Similarly, we also observed IFI16-IFI16 localization (red dots) in the nucleus and cytoplasm which was restricted only to the nucleus in the presence of C646 (B). (C and D) Quantitation of PLA spots of IFI16 with STING and H2B with STING in KSHV *de novo* infection. Uninfected and KSHV infected (4 h) HMVEC-d cells were fixed, permeabilized and tested by PLA using anti-IFI16 (mouse), STING (rabbit) and H2B (goat) antibodies as in S1A Fig. The average number of dots per cell in the nucleus and cytoplasm was quantitated and presented in the bar diagram. Magnification: 40X. PLA revealed the association of IFI16 with STING and between H2B and STING in KSHV infected cells. (E-H) UV-inactivated KSHV induced association of H2B-STING and IFI16-STING during *de novo* infection. Uninfected and KSHV (Live) or UV-inactivated KSHV (UV-KSHV) [2] infected (30 DNA copies/cell) HMVEC-d cells were fixed, permeabilized and subjected to PLA analysis using anti-H2B (goat), anti-STING (rabbit) and anti-IFI16 antibodies. PLA analysis revealed that UV-KSHV induced the association of H2B-STING or IFI16-STING similar to that of live-KSHV infection (E and F). Quantitation of H2B-STING or IFI16-STING average PLA spots per cell during live and UV-KSHV infection (G and H). (I) Quantitation of PLA spots of H2B with STING during KSHV latent infection (from Fig 5N). BJAB and BCBL-1 were fixed, permeabilized and subjected to PLA reactions using anti-H2B (goat) and anti-STING (rabbit) antibodies as described in S1A Fig. The average number of dots per cell in the nucleus and cytoplasm was quantitated and presented in the bar diagram. PLA analysis revealed the association of H2B with STING only in the cytoplasm of BCBL-1 cells.
(TIF)

S6 Fig Specificity controls for PLA, IP of STING during KSHV infection and HSV-1 infection of cells with knockdown of IFI16, BRCA1, H2B, cGAS, STING and ASC. (A and B) Specificity controls for PLA reactions. BJAB and BCBL-1 cells were fixed, permeabilized (as described in S1A Fig) and tested for PLA using only single species primary antibody, anti-H2B (A) and anti-IFI16 (B). DAPI was used as nucleus counter stain and the boxed areas of BCBL-1 cells were enlarged. Results showed no detection of any amplified dots which served as negative controls. Magnification: 40X. (C) Immunoprecipitation of STING with H2A and ASC during KSHV *de novo* infection. Cellular lysates (WCL) from HMVEC-d cells infected by KSHV for 4 h were immunoprecipitated using anti-STING and anti-ASC antibodies and immunoblotted for H2A and STING. The results showed no interaction of STING with H2A and between ASC and STING. (D and E) HMVEC-d cells infected with KSHV for 4 h were tested by PLA using primary antibodies, anti-STING (rabbit), anti-H2A (mouse) and anti-ASC (mouse) as described earlier (S1 Fig). PLA results revealed no localization of STING with H2A and ASC in KSHV infected and uninfected cells. (F) Effect of IFI16, H2B, BRCA1, cGAS, STING and ASC knockdown during HSV-1 infection. HFF cells were electroporated with siC, siIFI16, siBRCA1, siASC, siH2B, sicGAS and siSTING for 48 h followed by with/without HSV-1 infection (4 h). WCL were subjected to western blot analysis and results showed efficient knockdown of the above proteins.
(TIF)

S7 Fig Analysis of EdU labeled KSHV or HSV-1 genome DNA associated host cell proteins by chromatin pull down assay. (A and B) HMVEC-d and HFF cells were infected by unlabeled or EdU labeled KSHV genome (200 DNA copies/cell) or HSV-1 (10 pfu/cell) for 2 h and then protein-DNA cross-linking was performed. Biotin-TEG azide was selectively linked to the reactive alkyne group of EdU containing DNA via Click reaction. DNA was sheared and chromatin fragments were captured on streptavidin beads. The purified DNA from input or from pulled down samples was analyzed by agarose gel electrophoresis. DNA purified from unlabeled or EdU labeled- KSHV or HSV-1 infected cells showed similar levels (lanes 1 and 2). Streptavidin captured DNA was recovered only from cells infected with EdU-labeled virus (lane 4) but not from those infected with unlabeled virus (lane 3). These results confirm the specificity of the EdU genome pull down method. (C) Detection of HSV-1 genome associated host cell proteins by chromatin pull down during EdU-labeled virus infection. HFF cells electroporated with siC, siBRCA1 and siH2B for 48 h followed by infection with unlabeled or EdU labeled HSV-1 (10 pfu/cell) for 2 h and processed as described above. The purified DNA from input or pulled down samples was analyzed by agarose gel electrophoresis. Similar levels of DNA in unlabeled or EdU-labeled HSV-1 infected cells electroporated with siC, siBRCA1 and siH2B were observed (lanes 8–13). Recovered DNA by streptavidin captured materials was observed only from EdU-labeled HSV-1 infected cells (lanes 5–7) but not from unlabeled virus infected cells (lanes 2–4).
(TIF)
