# Supplementary material for: Histone H2B-IFI16 Recognition of Nuclear Herpesviral Genome Induces Cytoplasmic Interferon-β Responses
Source: PLoS Pathog. 2016 Oct 20;12(10):e1005967. doi: 10.1371/journal.ppat.1005967 (PMC5072618; doi:10.1371/journal.ppat.1005967)
Supplement: S1 Table — Nuclear fractions from uninfected HMVEC-d cells were isolated using a Nuclear Complex Co-IP Kit (Active Motif, CA). 100 μg of nuclear fraction was immunoprecipitated overnight at 4°C with anti-IFI16 or IgG control antibodies. Immunoprecipitates were resolved using a 4–20% gradient SDS-PAGE gel (Bio-Rad) and were stained with coomassie brilliant blue (CBB) dye. The bands of interest were analyzed by mass spectrometry (MS) using an LC-ESI (electrospray ionization)-MS based approach at the Midwest Proteome Center, Rosalind Franklin University of Medicine and Sciences. MS analysis revealed several proteins and the six proteins with the highest percentage of PEAKS scores and coverage are shown. (DOCX) [file ppat.1005967.s001.docx]

**S1 Table.** Proteins identified by mass spectrometric analysis of immunoprecipitates by anti-IFI16 and IgG control antibodies with nuclear lysates of uninfected HMVEC-d cells.

| **Proteins identified** | **PEAKS Score %** | **Coverage (%)** | **Mass** |
| --- | --- | --- | --- |
| Histone H2B | 96.9 | 23.81 | 13936.159 |
| Histone H4 | 60.5 | 8.82 | 11237.132 |
| Heterogeneous nuclear  ribonucleoprotein U isoform a | 73.5 | 4.48 | 90584.625 |
| Chain A, Solution Structure of the Rrm of Srp | 60.7 | 9.33 | 16820.625 |
| Chain A, NMR Structure of the Nalp1 pyrin domain Chain A, Solution Structure of the Rrm of Srp20 | 60.7 | 8.81 | 17656.602 |
| Bound to the RNA Cauc | 60.7 | 9.33 | 16834.65 |
