# Supplementary figures and images for: Histone H2B-IFI16 Recognition of Nuclear Herpesviral Genome Induces Cytoplasmic Interferon-β Responses
Source: PLoS Pathog. 2016 Oct 20;12(10):e1005967. doi: 10.1371/journal.ppat.1005967 (PMC5072618; doi:10.1371/journal.ppat.1005967)

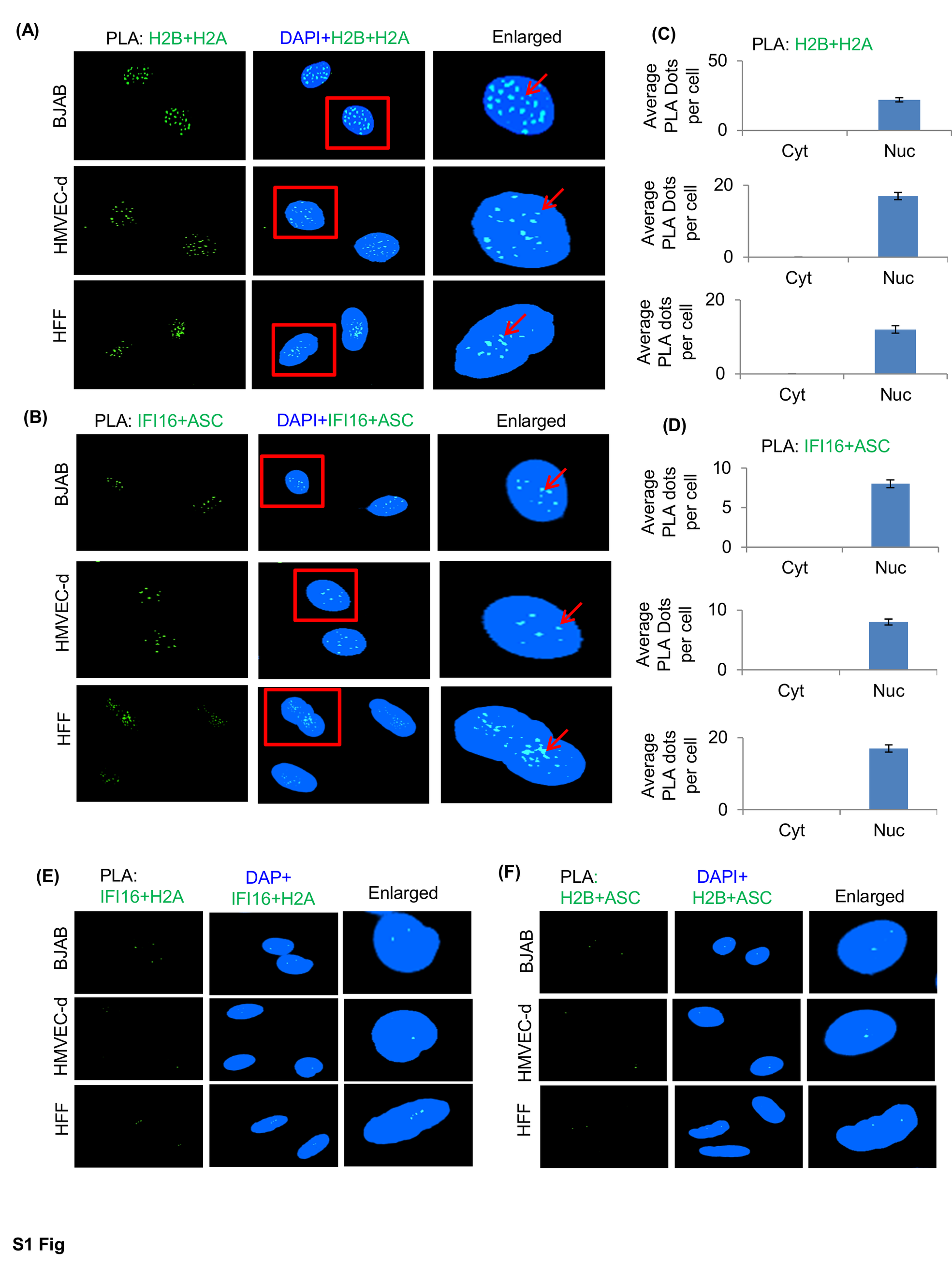

Supplement: S1 Fig — (A and B) Protein-protein close proximity interactions were detected by a DUOLink PLA kit (Sigma). Uninfected BJAB cells were washed with PBS by centrifugation at 200xg at 4°C and spotted on 10-well glass slides, fixed, permeabilized with pre-chilled acetone, and blocked with DUOLink blocking buffer for 30 min at 37°C. Uninfected HMVEC-d and HFF cells cultured in 8 well chamber microscope slides were fixed, permeabilized and blocked with DUOLink blocking buffer for 30 min at 37°C. Blocked BJAB, HMVEC-d and HFF cells were incubated with primary antibodies, anti-H2B (rabbit), anti-H2A (mouse), anti-IFI16 (rabbit) or anti-ASC (mouse) antibodies for 1 h at 37°C, washed, incubated for 1 h at 37°C with species specific PLA probes (PLUS and MINUS probes), anti-mouse probe (+) and anti-rabbit probe (-), under hybridization conditions in the presence of two additional oligonucleotides to enable hybridization of PLA probes that were in close proximity (<40 nm). A ligation mixture with ligase was added to link the two hybridized oligonucleotides to form a closed circle. Multiple cycles of rolling-circle amplification using the ligated circle as a template were performed by adding an amplification solution to form a concatemeric product extending from the oligonucleotide arm of the PLA probe. Eventually, a detection solution containing fluorescently labeled oligonucleotides was added to hybridize with the concatemeric products. The signal was detected as a distinct fluorescent dot in the Texas red or FITC green channel depending on the probes and analyzed by fluorescence microscopy. The association of H2B with H2A and IFI16 with ASC was observed by green colored dots in the nucleus of the above cells as indicated by red arrows. Nuclei were stained by DAPI and boxed areas were enlarged in the rightmost panels. (C and D) Bar diagrams represent the quantitation of the average number of PLA dots per cell in the cytoplasm and nucleus of uninfected BJAB, HMVEC-d and HFF cells. (E and [file ppat.1005967.s002.tif]

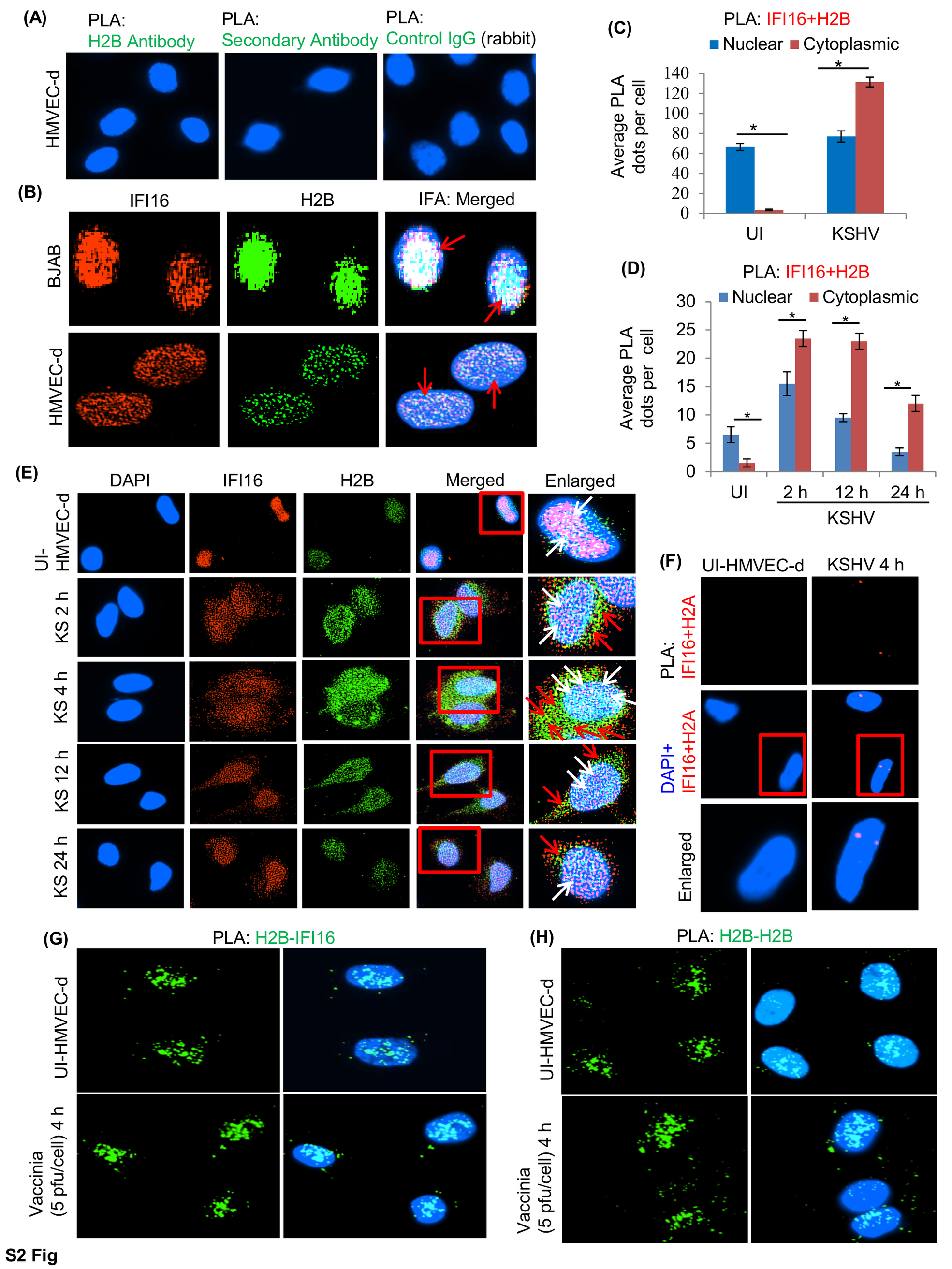

Supplement: S2 Fig — (A) Specificity controls for PLA reactions. As specificity controls for all PLA reactions, negative controls such as use of a single species primary antibody, secondary antibody alone or control IgG antibody were used to perform the complete PLA process as described in S1A Fig. Magnification: 40X. (B) Localization of IFI16 with H2B by IFA. BJAB and HMVEC-d cells were fixed, permeabilized, blocked in Image-iT signal enhancer, incubated with primary anti-IFI16 and anti-H2B antibodies for 1 h. After washing, these were incubated with secondary antibodies, anti-mouse Alexa Fluor 594 for IFI16 and anti-rabbit Alexa Fluor 488 for H2B, for 1 h. DAPI was used for nuclear staining. Boxed areas were enlarged in the rightmost panels. Red arrows indicate the colocalization of IFI16 with H2B in the nucleus. (C and D) Quantitation of PLA spots of IFI16-H2B during KSHV de novo infection. Uninfected HMVEC-d cells were infected for 4 h (C) and 2, 12 and 24 h (D) with KSHV (30 DNA copies/cell) and subjected to PLA reaction using anti-IFI16 (mouse) and H2B (rabbit) antibodies as described in S1A Fig. PLA analysis revealed the association of IFI16 with H2B during KSHV de novo infection. The average number of spots per cell in the nucleus and cytoplasm was quantitated and presented in the bar diagram. Magnification: 40X. (E) Localization of IFI16 with H2B during KSHV (KS) de novo infection by IFA. HMVEC-d cells were infected by KSHV (30 DNA copies/cell) for 2 h, washed and then incubated in complete medium for various time points (2, 4, 12, 24 h). Uninfected and KSHV infected cells were fixed, permeabilized, blocked, incubated with anti-IFI16 and anti-H2B primary antibodies for 1 h at RT, followed by incubation with secondary antibodies (IFI16:anti-mouse Alexa Fluor 594; H2B:anti-rabbit Alexa Fluor 488) for 1 h. DAPI was used as nuclear stain and the boxed areas from the merged panels were enlarged in the rightmost panels. White and red arrows represent localization of IFI16 with H2B in [file ppat.1005967.s003.tif]

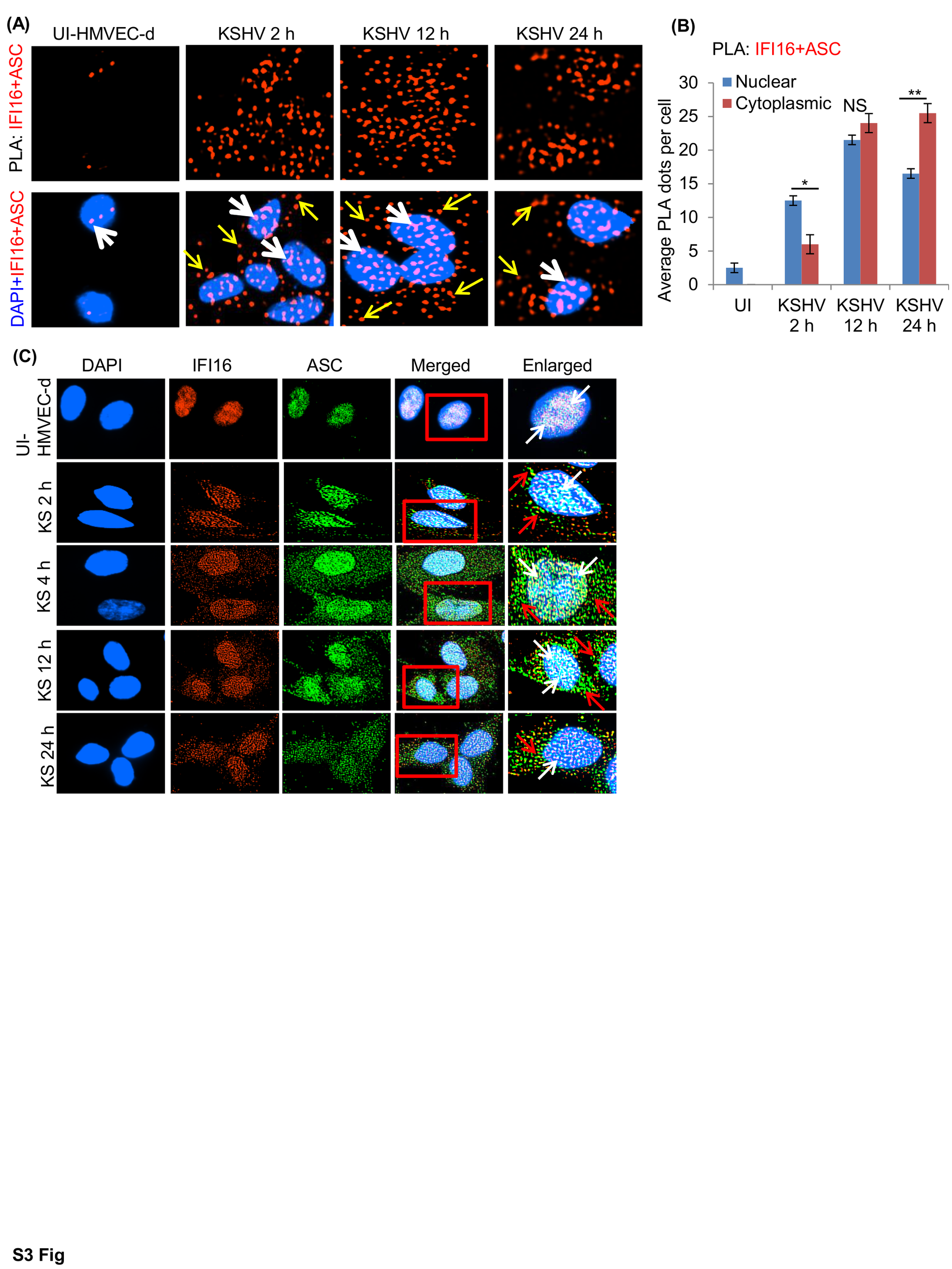

Supplement: S3 Fig — (A) HMVEC-d cells were infected by KSHV for 2, 12 and 24 h and PLA reaction was completed using anti-IFI16 and anti-ASC primary antibodies. PLA results showed a few red dots of IFI16-ASC in the nucleus of uninfected cells which profoundly increased in the nucleus as well as in the cytoplasm of cells at 2, 12 and 24 h post-KSHV infection. (B) Average numbers of FI16-ASC PLA red dots in the nucleus and cytoplasm per cell were quantitated and presented in the bar graph. Nucleus vs. cytoplasm dots statistics: * p<0.05, ** p<0.01, NS: not significant. (C) Localization of IFI16 with ASC during KSHV (KS) de novo infection by IFA. HMVEC-d cells were infected by KSHV (30 DNA copies/cell), permeabilized as described in S2E Fig, immunostained with anti-IFI16 and anti-ASC primary antibodies for 1 h at RT, and followed by incubation with secondary antibodies (IFI16-anti-mouse Alexa Fluor 594; ASC-anti-goat Alexa Fluor 488). DAPI was used as nuclear stain and the boxed areas from merged panels were enlarged in the rightmost panels. White and red arrows represent localization of IFI16 with ASC in the nucleus and cytoplasm, respectively. Magnification: 40X. (TIF) [file ppat.1005967.s004.tif]

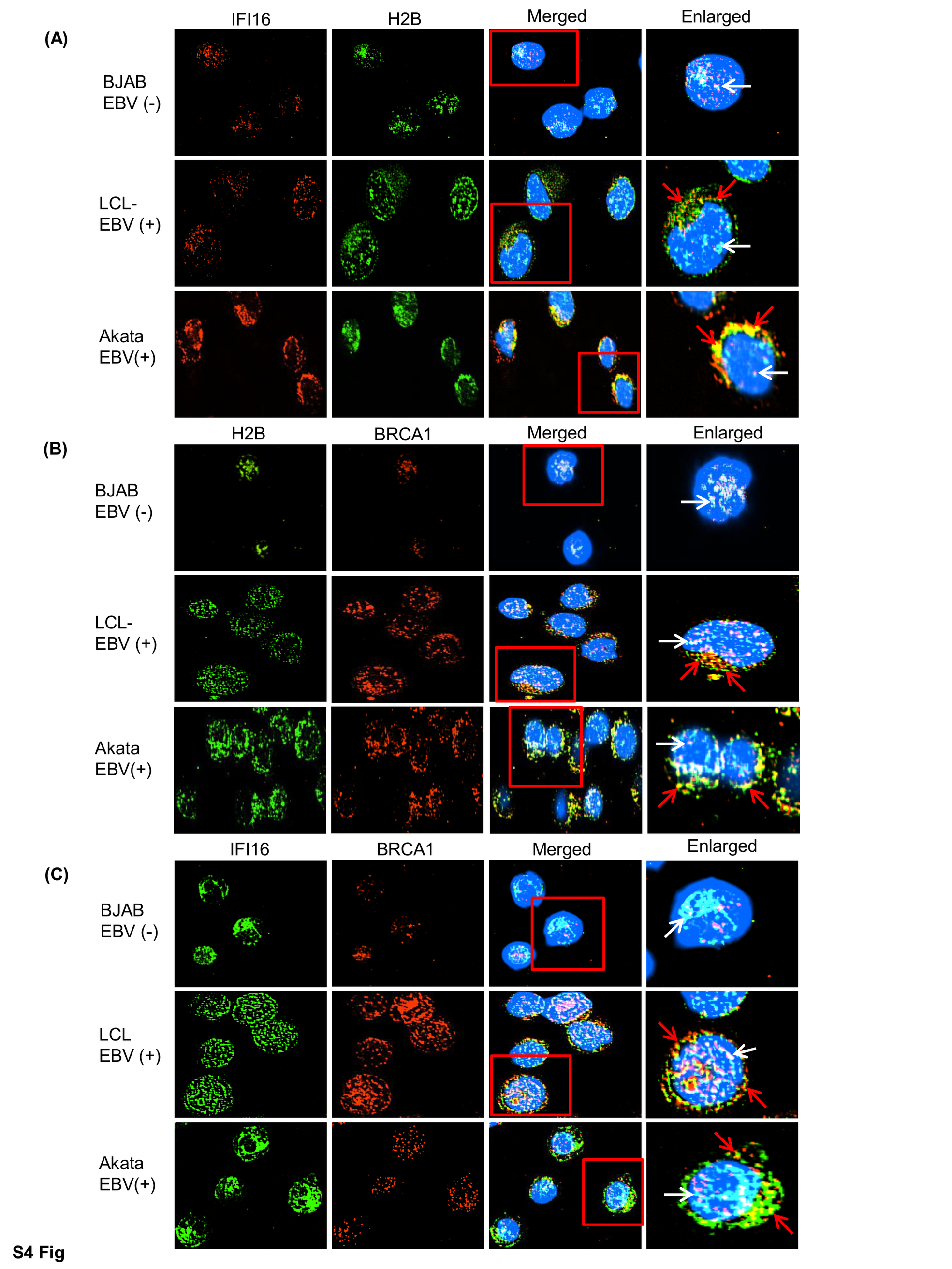

Supplement: S4 Fig — (A-C) EBV (-) BJAB and EBV (+) LCL (latency III) and EBV (+) Akata (latency I) cells were washed with PBS, spotted on glass slides, fixed/permeabilized with pre-chilled acetone, blocked using Image-iT signal enhancer for ~20 min, incubated with primary anti-IFI16 (mouse), anti-H2B (rabbit or goat) or anti-BRCA1 (rabbit) antibodies and then secondary antibodies as described in S2E Fig. DAPI was used as nuclear stain and the boxed areas from merged panels were enlarged in the rightmost panels. White and red arrows represent localization in the nucleus and cytoplasm, respectively. Magnification: 40X. (TIF) [file ppat.1005967.s005.tif]

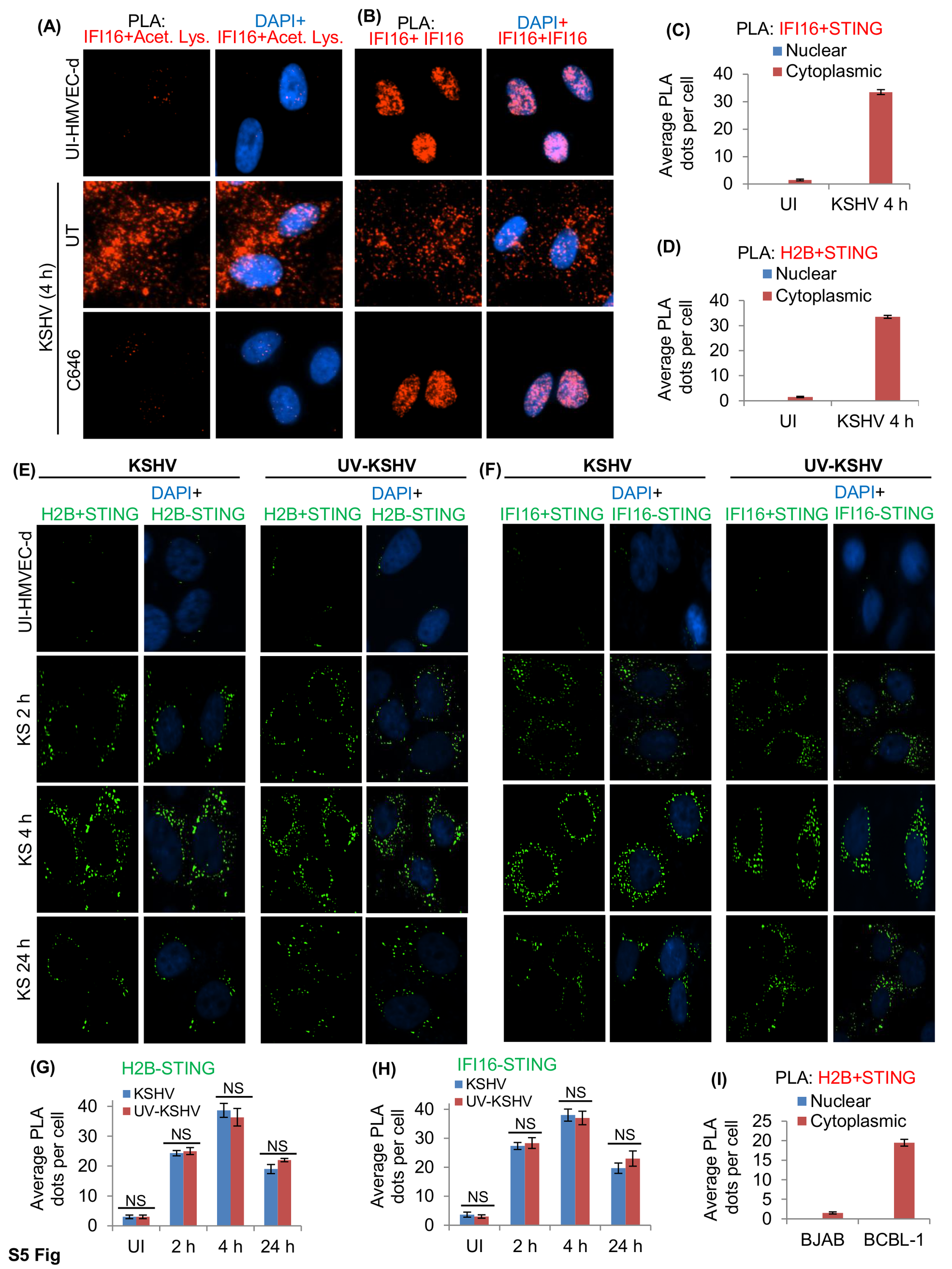

Supplement: S5 Fig — (A and B) PLA analysis for the detection of IFI16 acetylation during KSHV de novo infection. Untreated (UT) HMVEC-d cells or cells pre-incubated with p300 inhibitor C646 (1 μM) for 2 h were infected with KSHV (30 DNA copies/cell) for 2 h, washed, and incubated with complete medium for 2 more hours with or without C646. These cells were processed for PLA reactions as in S1 Fig using anti-acetyl lysine (rabbit) and anti-IFI16 (mouse or rabbit) antibodies. Compared to uninfected cells, PLA analysis revealed substantial localization of IFI16 with acetyl lysine in the nucleus and cytoplasm (red dots) of KSHV infected (UT-untreated) cells which was significantly reduced in the presence of C646 (A). Similarly, we also observed IFI16-IFI16 localization (red dots) in the nucleus and cytoplasm which was restricted only to the nucleus in the presence of C646 (B). (C and D) Quantitation of PLA spots of IFI16 with STING and H2B with STING in KSHV de novo infection. Uninfected and KSHV infected (4 h) HMVEC-d cells were fixed, permeabilized and tested by PLA using anti-IFI16 (mouse), STING (rabbit) and H2B (goat) antibodies as in S1A Fig. The average number of dots per cell in the nucleus and cytoplasm was quantitated and presented in the bar diagram. Magnification: 40X. PLA revealed the association of IFI16 with STING and between H2B and STING in KSHV infected cells. (E-H) UV-inactivated KSHV induced association of H2B-STING and IFI16-STING during de novo infection. Uninfected and KSHV (Live) or UV-inactivated KSHV (UV-KSHV) [2] infected (30 DNA copies/cell) HMVEC-d cells were fixed, permeabilized and subjected to PLA analysis using anti-H2B (goat), anti-STING (rabbit) and anti-IFI16 antibodies. PLA analysis revealed that UV-KSHV induced the association of H2B-STING or IFI16-STING similar to that of live-KSHV infection (E and F). Quantitation of H2B-STING or IFI16-STING average PLA spots per cell during live and UV-KSHV infection (G and H). (I) Quantitation of PLA spots of H2B wi [file ppat.1005967.s006.tif]

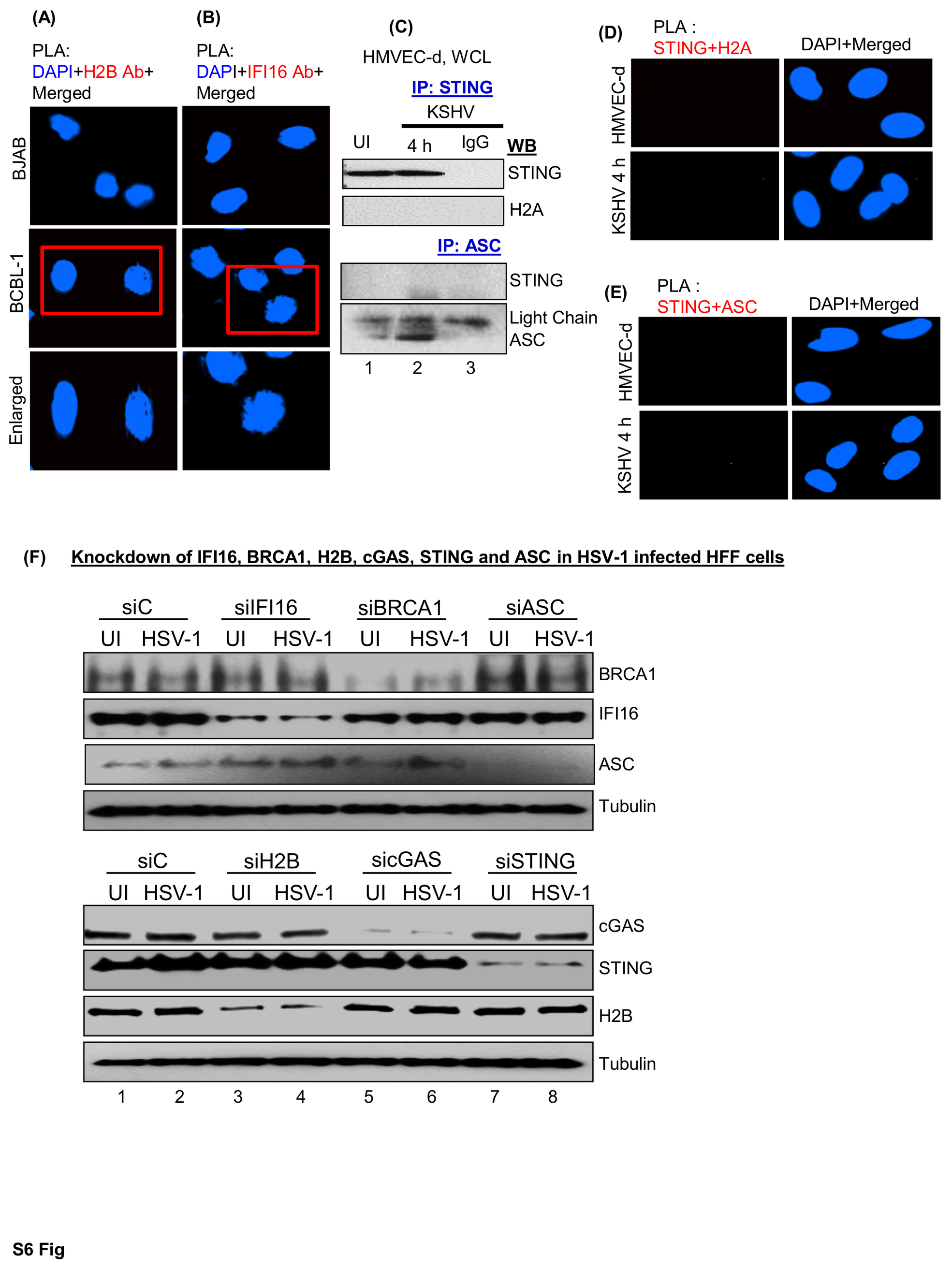

Supplement: S6 Fig — (A and B) Specificity controls for PLA reactions. BJAB and BCBL-1 cells were fixed, permeabilized (as described in S1A Fig) and tested for PLA using only single species primary antibody, anti-H2B (A) and anti-IFI16 (B). DAPI was used as nucleus counter stain and the boxed areas of BCBL-1 cells were enlarged. Results showed no detection of any amplified dots which served as negative controls. Magnification: 40X. (C) Immunoprecipitation of STING with H2A and ASC during KSHV de novo infection. Cellular lysates (WCL) from HMVEC-d cells infected by KSHV for 4 h were immunoprecipitated using anti-STING and anti-ASC antibodies and immunoblotted for H2A and STING. The results showed no interaction of STING with H2A and between ASC and STING. (D and E) HMVEC-d cells infected with KSHV for 4 h were tested by PLA using primary antibodies, anti-STING (rabbit), anti-H2A (mouse) and anti-ASC (mouse) as described earlier (S1 Fig). PLA results revealed no localization of STING with H2A and ASC in KSHV infected and uninfected cells. (F) Effect of IFI16, H2B, BRCA1, cGAS, STING and ASC knockdown during HSV-1 infection. HFF cells were electroporated with siC, siIFI16, siBRCA1, siASC, siH2B, sicGAS and siSTING for 48 h followed by with/without HSV-1 infection (4 h). WCL were subjected to western blot analysis and results showed efficient knockdown of the above proteins. (TIF) [file ppat.1005967.s007.tif]

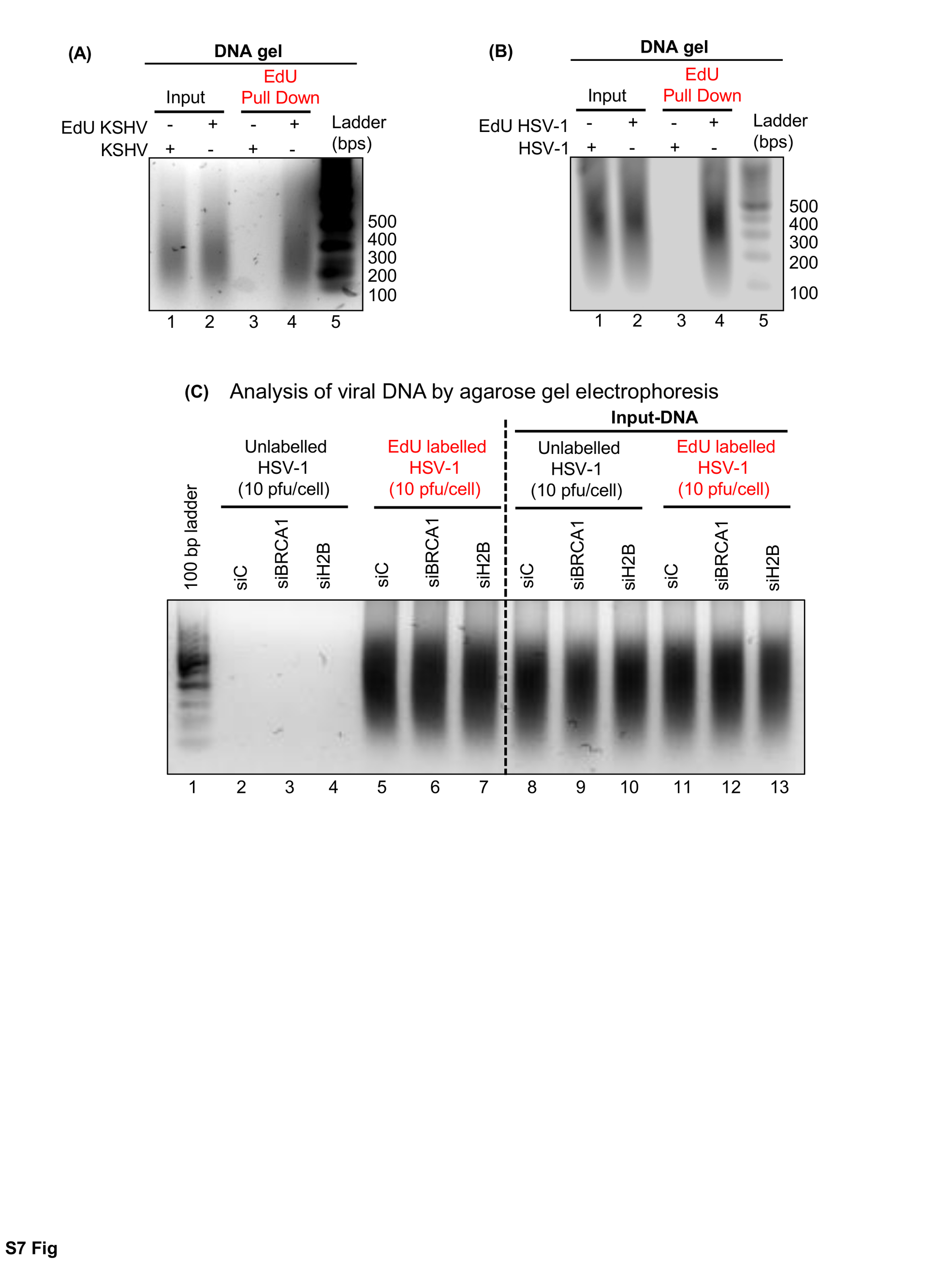

Supplement: S7 Fig — (A and B) HMVEC-d and HFF cells were infected by unlabeled or EdU labeled KSHV genome (200 DNA copies/cell) or HSV-1 (10 pfu/cell) for 2 h and then protein-DNA cross-linking was performed. Biotin-TEG azide was selectively linked to the reactive alkyne group of EdU containing DNA via Click reaction. DNA was sheared and chromatin fragments were captured on streptavidin beads. The purified DNA from input or from pulled down samples was analyzed by agarose gel electrophoresis. DNA purified from unlabeled or EdU labeled- KSHV or HSV-1 infected cells showed similar levels (lanes 1 and 2). Streptavidin captured DNA was recovered only from cells infected with EdU-labeled virus (lane 4) but not from those infected with unlabeled virus (lane 3). These results confirm the specificity of the EdU genome pull down method. (C) Detection of HSV-1 genome associated host cell proteins by chromatin pull down during EdU-labeled virus infection. HFF cells electroporated with siC, siBRCA1 and siH2B for 48 h followed by infection with unlabeled or EdU labeled HSV-1 (10 pfu/cell) for 2 h and processed as described above. The purified DNA from input or pulled down samples was analyzed by agarose gel electrophoresis. Similar levels of DNA in unlabeled or EdU-labeled HSV-1 infected cells electroporated with siC, siBRCA1 and siH2B were observed (lanes 8–13). Recovered DNA by streptavidin captured materials was observed only from EdU-labeled HSV-1 infected cells (lanes 5–7) but not from unlabeled virus infected cells (lanes 2–4). (TIF) [file ppat.1005967.s008.tif]
